# An Optimal Control Model to Understand the Potential Impact of the New Vaccine and Transmission-Blocking Drugs for Malaria: A Case Study in Papua and West Papua, Indonesia

**DOI:** 10.3390/vaccines10081174

**Published:** 2022-07-24

**Authors:** Bevina D. Handari, Rossi A. Ramadhani, Chidozie W. Chukwu, Sarbaz H. A. Khoshnaw, Dipo Aldila

**Affiliations:** 1 Department of Mathematics, Universitas Indonesia, Depok 16424, Indonesia; bevina@sci.ui.ac.id (B.D.H.); rossi.alya@sci.ui.ac.id (R.A.R.); 2 Division of Infectious Diseases and Global Public Health, University of California, San Diego, CA 94720, USA; wiliam.chukwu@gmail.com; 3 Department of Mathematics, University of Raparin, Ranya 46012, Kurdistan Region, Iraq; sarbaz.hamza@uor.edu.krd

**Keywords:** malaria, vaccine, treatment, controlled reproduction number, optimal control

## Abstract

Malaria is one of the major causes of a high death rate due to infectious diseases every year. Despite attempts to eradicate the disease, results have not been very successful. New vaccines and other treatments are being constantly developed to seek optimal ways to prevent malaria outbreaks. In this article, we formulate and analyze an optimal control model of malaria incorporating the new pre-erythrocytic vaccine and transmission-blocking treatment. Sufficient conditions to guarantee local stability of the malaria-free equilibrium were derived based on the controlled reproduction number condition. Using the non-linear least square fitting method, we fitted the incidence data from the province of Papua and West Papua in Indonesia to estimate the model parameter values. The optimal control characterization and optimality conditions were derived by applying the Pontryagin Maximum Principle, and numerical simulations were also presented. Simulation results show that both the pre-erythrocytic vaccine and transmission-blocking treatment significantly reduce the spread of malaria. Accordingly, a high doses of pre-erythrocytic vaccine is needed if the number of infected individuals is relatively small, while transmission blocking is required if the number of infected individuals is relatively large. These results suggest that a large-scale implementation of both strategies is vital as the world continues with the effort to eradicate malaria, especially in endemic regions across the globe.

## 1. Introduction

The primary cause of any infectious disease is the spread of pathogens such as bacteria, viruses, parasites, or fungus. Some of these diseases can spread from person to person, either through direct or indirect contact. A disease that spreads in the human population with an intermediate vector is called a vector-borne disease. According to the world health organization (WHO) [1], vector-borne infection contributes to over 17% of total infectious disease cases worldwide, with an estimated 700,000 deaths annually. Malaria is a vector-borne disease that threatens millions of people every year. Most of the cases come from Africa, southeast Asia, the eastern Mediterranean, and West Pacific [2]. Globally, there were over 600,000 deaths reported out of over 240 million detected cases in 2020. In Indonesia, over 74% of all detected cases are from the provinces of Papua and West Papua [3].

Malaria spreads through the bite of female Anopheles mosquitoes from previously infected individuals with *Plasmodium* (P). The five types of *Plasmodium* that cause malaria are *P. falciparum, P. vivax, P. malariae, P. ovale*, and *P. knowlesi* [4]. Globally, among all these types of *Plasmodium*, *P. falciparum* is primarily commonly found in African countries, while *P.vivax* exists in other parts of the world (that is, outside Africa) [5]. Several forms of intervention to control the spread of malaria have been introduced for many years worldwide. Most forms of intervention aim to control the population of mosquitoes in the field, such as with the use of fumigation, larvacide, or insecticide-treated bed nets [6]. Other than vector control, malaria prevention also focuses on developing a new vaccine to prevent new infections in the human population. Based on the life cycle of *Plasmodium* in the human body, the malaria vaccine is divided into three types, namely pre-erythrocytic vaccine, blood-stage vaccine, and transmission-blocking vaccine [7]. The pre-erythrocytic vaccine was designed to eliminate sporozoites immediately after mosquitoes inject *Plasmodium* into the human body. It will also block the *Plasmodium* from going to the human liver. According to the WHO report [5], RTS, S/AS01 is one type of pre-erythrocytic vaccine that is recommended by WHO to kill *P. falciparum* in children population. The blood-stage vaccine is introduced to target the asexual stage of *Plasmodium* (merozoites) in human red blood cells. On the other hand, the transmission-blocking vaccine is given such that further infection can be avoided [8]. Other than the transmission-blocking vaccine, a type of transmission-blocking drug has been used to eliminate the sexual stages of *Plasmodium* in human or mosquito bodies.

Mathematical models have been used for many years to understand the mechanisms of malaria transmission, since such model approaches can be used to give a visual interpretation of any possible intervention that can be implemented in the field to control malaria transmission. These approaches provide a scientific background before a final decision from the government should be taken. From the early work by Ross [9] in 1911, many mathematical models were introduced by authors to help a better understanding of malaria transmission. Macdonald in 1957 [10] used his model to estimate the infection and recovery rates of malaria. He found that reducing the number of mosquitoes in an endemic area is an inefficient malaria control strategy. In the early 1980s–1990s, Anderson and May [11] and Aron and May [12] constructed their malaria model based on the assumption that immunity to malaria is independent of the duration of exposure. Okosun et al., in [13], concluded from their mathematical model that a combination between insecticides and transmission-blocking treatment is the most cost-effective interventions to control malaria. An optimal vaccination and bed net mathematical model have been introduced by Prosper et al. in [14]. Their analytical result reveals that increasing the case detection strategy may reduce the chance of backward bifurcation phenomena in their model. An analysis of the potential impact of pre-erythrocytic vaccine from clinical data was discussed by the author in [15]. Similar to Prosper et al. in [14], Woldegerima et al. in [16] also found a possible backward bifurcation from their model on the impact of transmission-blocking drugs. Their model projects an approximately 82% death rate is reduced by 2035 if 35% of the population in Sub-Saharan Africa receives a transmission-blocking drug with an efficacy of 95%. Kuddus and Rahman in [17] analyze the dynamics of malaria using incidence data in Bangladesh from 2001 to 2014. They found that as infection rate has the greatest impact on the basic reproduction number compared to other model parameters, it is important to reduce this infection rate, such as using insecticide-treated bed nets, spraying insecticides, clearing stagnant water, etc. In a more recent mathematical model, authors include more recent facts on malaria transmission such as the effect of vector-bias [18], asymptotic carriers [19], age-structured [20], competitive strains [21], seasonal factor [22,23], and coinfection of malaria with COVID-19 [24]. Furthermore, intervention models also have been widely introduced by authors, such as the use of fumigation [25], insecticide-treated bed nets [26], and vaccines with waning immunity [27], or transmission-blocking vaccines [28].

The authors in [29] concluded that a better understanding of *Plasmodium* development in the pre-erythrocytic stage could lead to the discovery of new antigenic targets for enhanced immunization strategies. These findings may aid in the development of new malaria immunization techniques that are more successful and useful. In 2015, GlaxoSmithKline’s (GSK’s) RTS, S/AS01 pre-erythrocytic vaccine received a positive scientific response on the quality of this vaccine in combating malaria transmission [30]. Based on the above description, we consider it essential to see the potential impact of the pre-erythrocytic vaccine (RTS, S/AS01) and transmission-blocking drug as a combination of control to reduce the spread of malaria using a mathematical model approach.

Our model divided the susceptible population based on a condition, whether they use a pre-erythrocytic vaccine or not. Furthermore, we also consider the possibility that the transmission-blocking drug cannot kill all *Plasmodium* in either the sexual or asexual stage in the human body. We used incidence data from Papua and West Papua to calibrate our model by determining the best-fit parameter on our model. Sensitivity analysis on the basic reproduction number and the numerical simulation on the dynamics of each compartment are also conducted to see the impact of pre-erythrocytic vaccine and transmission-blocking drugs in the malaria control strategy. An optimal control model is then analyzed to determine the best possible scenario for combining pre-erythrocytic vaccine and transmission-blocking drugs. The novelty of our model lies in the originality of the model, where we discuss the potential impact of the pre-erythrocytic vaccine. Furthermore, we also use incidence data from two provinces in Indonesia, namely Papua and West Papua, to calibrate our model parameters. These incidence data have never been used in any malaria mathematical model.

The organization of this paper is as follows. In Section 2, we construct our model based on our assumptions, and estimate the parameter values on our model by fitting the output of our model with incidence data in Papua and West Papua. Dynamical analysis regarded the positiveness of the solution of our model, the existence and stability of equilibrium points, and the controlled reproduction number discussed in Section 3. Sensitivity analysis is given in Section 4. Existence of optimal solution and the characterization of the optimal control problem are given in Section 5 and Section 6, respectively. Finally, some conclusions are given in Section 7.

## 2. The Model and Parameter Estimation Result

### 2.1. The Model

Based on the malaria status in the human and mosquitoes population, we divided the total human population (denoted by *N*) into eight compartments as follows:

1. Susceptible without vaccine ($S_{1}$) consists of a group of individuals susceptible to malaria who have not received a pre-erythrocytic vaccine yet.
2. Susceptible with a vaccine ($S_{2}$) consists of a group of individuals who are also susceptible to malaria but have already received a pre-erythrocytic vaccine.
3. Exposed without vaccine $\left(E_{1}\right)$ consists of a group of newly infected individuals from $S_{1}$ who have not yet gotten the pre-erythrocytic vaccine. We assume that individuals in this compartment are in the leaver stage. Hence, although these individuals do not show any symptoms yet, we assume that they can transmit *Plasmodium* to susceptible mosquitoes.
4. Exposed with vaccine $\left(E_{2}\right)$ consists of a group of newly infected individuals from $S_{2}$. Although these individuals have already gotten vaccinated, the description is still the same with $E_{1}$.
5. Infected (I) consists of a group of individuals who have already gotten infected by malaria and show their symptoms.
6. Infected individuals undergo transmission-blocking treatment (T), defined as a group of individuals who already get infected, show symptoms, but get a transmission-blocking treatment. We assume that this treatment can kill the sexual *Plasmodium* (gametocytes).
7. Recovered but carrier $\left(R_{1}\right)$ consists of individuals who recovered from malaria (do not show symptoms anymore) and have a temporal immunity but still have asexual *Plasmodium* inside their bodies. Hence, this group of individuals can still transmit *Plasmodium* to the mosquito.
8. Fully recovered $\left(R_{2}\right)$ consists of a group of individuals who recovered from malaria and succeeded in the transmission-blocking treatment process. Hence, unlike in $R_{1}$, individuals in $R_{2}$ lack sexual and asexual *Plasmodium* in their blood. Therefore, $R_{2}$ compartment does not transmit malaria anymore.

On the other hand, we only divide the mosquitoes population (denoted by *M*) into two compartments, namely the susceptible and infected mosquitoes, denoted by *V* and *W*, respectively.

We use the following assumptions to construct the model:

1. The rate of new individuals only came from newborns with a constant rate of $\Pi_{h}$. We ignore migration from our model.
2. Vertical transmission is neglected [31].
3. The infected individuals in $E_{1},E_{2},I,T,$ and $R_{1}$ are capable to transmit *Plasmodium* to mosquitoes with a constant rate $\beta_{v}$.
4. The pre-erythrocytic vaccine is given to $S_{1}$ individuals with a constant rate $u_{1}$ to give temporal protection from mosquito bites that can lead to malaria infection. Hence, we assume that the transmission rate of $S_{2}$ is less than $S_{1}$ $\left(\beta_{h2}<\beta_{h1}\right)$.
5. The pre-erythrocytic vaccine is not for a lifetime. Hence, after a period of $\alpha^{-1}$, individuals in $S_{2}$ will return to $S_{1}$.
6. The transmission-blocking treatment is given to individuals in *I* with a constant rate $u_{2}$ to cure malaria and wipe out sexual and asexual *Plasmodium* from their blood.
7. The transmission-blocking treatment is not always successful in curing infected individuals.
8. The description of all parameters is given in Table 1 and assumed to be nonnegative.

**Figure 1:**
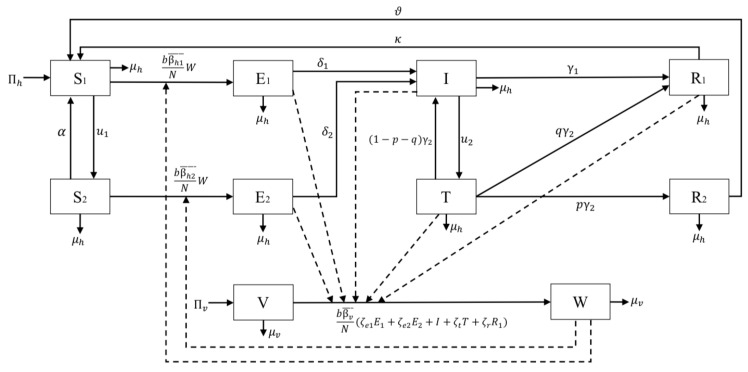
Transmission diagram of model (1).

The construction of the model is based on the transmission diagram in Figure 1. All newborns in human and mosquito populations are assumed to be susceptible, with a rate of $\Pi_{h}$ and $\Pi_{v}$, respectively. On the other hand, we assume that each compartment has a natural death rate of $\mu_{h}$ and $\mu_{v}$ for human and mosquito populations, respectively. According to [32,33], the total inhabitants in Papua and West papua are 3,453,430 and 981,822, respectively.

Our main purpose is to understand the potential impact of the pre-erythrocytic vaccine on reducing the possible transmission of malaria in the human population. This vaccine was designed to clean the sporozoite from the human body right after the mosquito injects the *Plasmodium* into the human body and block the sporozoites’ invasion of the human liver cell. Hence, we include the rate of this pre-erythrocytic with a constant rate of $u_{1}$ to the susceptible population $\left(S_{1}\right)$. This vaccine is not for a lifetime [37]. Hence, after a certain period of time, individuals in $S_{2}$ will return to $S_{1}$. We denote this dropout rate with α. The transmission of malaria comes from the bite of infected mosquitoes to susceptible humans. We assume that the successful transmission rates of $S_{1}$ and $S_{2}$ are denoted by $\bar{\beta_{h1}}$ and $\bar{\beta_{h2}}$, respectively. Let *b* be the average number of mosquitoes bite per month, then $b\bar{\beta_{h1}}$ and $b\bar{\beta_{h2}}$ are the averages of possible success transmission in $S_{1}$ and $S_{2}$ each month due to the bite of one infected mosquitoes, respectively. We use a ratio-dependent function to model our infection term. Therefore, the number of total infections in $S_{1}$ is given by $\frac{b\bar{\beta_{h1}}WS_{1}}{N}$. We assume that the total human population is constant. Therefore, $\frac{b\bar{\beta_{h1}}}{N}$ is constant, and we denote it by $\beta_{h1}$. Therefore, we have that the total number of new infections in the human population from the $S_{1}$ compartment is given by $\beta_{h1}S_{1}W$. Using the same approach, the number of new infections in $S_{2}$ is given by $\beta_{h2}S_{2}W$. Exposed individuals without vaccine $\left(E_{1}\right)$ and with vaccine $\left(E_{2}\right)$ move to Infected class (I) due to progression rate $\delta_{1}$ and $\delta_{2}$, respectively. Note that due to the effect of pre-erythrocytic vaccine that can suppress invasion of sporozoites in hepatocytes [8], we have $\delta_{2}<\delta_{1}$. Without treatment, we assume that infected individual *I* can recover with a constant rate $\gamma_{1}$.

Another essential factor that is considered in this article is the use of transmission-blocking treatment for infected individuals *I*. We assume that individuals in *I* get a treatment with a constant rate $u_{2}$, which will transfer them into compartment *T*. This treatment is given in a duration of $\gamma_{2}^{-1}$. Hence, after a period of $\gamma_{2}^{-1}$, the result of this treatment should be evaluated. If the treatment successfully kills all sexual and asexual parasites, then they are transferred to the compartment of $R_{2}$ with a rate of $p\gamma_{2}$. If the treatment only partially succeeds where only sexual parasites could be eliminated but not the asexual parasites, these individuals will go to $R_{1}$ with a rate of $q\gamma_{2}$. Finally, if the treatment fails, then individuals in *T* go back to *I* with a rate of $\left(1-p-q\right)\gamma_{2}$. We assume that individuals in $R_{1}$ still could infect mosquitoes, but they are immune to the symptoms of malaria. Further, we also assume that they have a temporal immunity of $\kappa^{-1}$. On the other hand, individuals in $R_{2}$ cannot transfer *Plasmodium* to mosquitoes and have a temporal immunity of $\vartheta^{-1}$.

Unlike the human population, the modeling of the mosquito population is not as complex. It only involves newborn $\Pi_{v}$, natural death rate $\mu_{v}$, and infection. We assume that susceptible mosquitoes will get infected if they bite infected individuals in compartments $E_{1},E_{2},I,T,$ and $R_{1}$. Since the status of parasites in each mentioned compartment is in different stages, we assume the transmission rate for the mosquitoes are different based on their parasites status. Gametocytes are sexual forms of parasites produced by blood-stage merozoites. However, for *P. vivax*, gametocytes can be produced by liver stage merozoites [39]. Therefore, humans still in the pre-erythrocyte stage can also transmit susceptible mosquitoes. Exposed humans who received the pre-erythrocytic vaccine $\left(E_{1}\right)$ will have fewer parasites in the liver, so the likelihood of liver-stage merozoites producing gametocytes will be less than that of exposed unvaccinated humans $\left(E_{2}\right)$. In addition, according to [39], gametocytes can still be found in peripheral blood after several weeks of asexual parasite infection being cleared, so recovered humans $\left(R_{1}\right)$ can still transmit susceptible mosquitoes. Humans in treatment can transmit susceptible mosquitoes because humans in treatment (T) are people who are still infected or have not been fully treated. Hence, the transmission rate of $E_{1},E_{2},R,T$ to susceptible mosquitoes are given by $\zeta_{e2}\bar{\beta_{v}},\zeta_{e1}\bar{\beta_{v}},\zeta_{r}\bar{\beta_{v}},$ and $\zeta_{t}\bar{\beta_{v}}$, respectively. Note that $\zeta_{e1},\zeta_{e2},\zeta_{r}$, and $\zeta_{t}$ are the correction parameters due to parasites’ stage in human body, and satisfies the following inequality:

$$0<\zeta_{e2}<\zeta_{e1}<\zeta_{r}<\zeta_{t}<1.$$

Therefore, the total of new infection in mosquitoes population is given by

$$\beta_{v}\left(\zeta_{e1}E_{1}+\zeta_{e2}E_{2}+I+\zeta_{r}R+\zeta_{t}T\right)V,$$

where $\beta_{v}=\frac{b\bar{\beta_{v}}}{N}$. From all mentioned parameters above, we must understand that some parameters, especially in the mosquito population, might depend on time or other factors such as weather. For example, mosquitoes are more active when the temperature is low, which means that mosquitoes will be more active in biting humans when the temperature is relatively low [40]. Based on this explanation, all parameter values in our model as in Table 1 are average values.

In many mathematical models for disease control, an optimal control approach is commonly used by many authors to understand the behavior of each intervention as a time-dependent variable under a specific budget limitation problem [41,42,43]. Our proposed model has two different forms of intervention: the pre-erythrocytic vaccine and transmission-blocking treatment, denoted by $u_{1}$ and $u_{2}$, respectively. In this article, these two forms of intervention will be treated as a time-dependent variable to pursue the best strategy to suppress the spread of malaria. Hence, we have $u_{1}=u_{1}\left(t\right)$ and $u_{2}=u_{2}\left(t\right)$.

Based on assumptions above, we describe the dynamics of malaria under the effect of pre-erythrocytic vaccine and transmission-blocking treatment by the following system of ordinary differential equations:

(1) $$\begin{array}{ccc} \frac{dS_{1}}{dt} & = & \Pi_{h}+\kappa R_{1}+\vartheta R_{2}+\alpha S_{2}-\beta_{h1}S_{1}W-\left(u_{1}\left(t\right)+\mu_{h}\right)S_{1},\\ \frac{dS_{2}}{dt} & = & u_{1}\left(t\right)S_{1}-\beta_{h2}S_{2}W-\left(\alpha +\mu_{h}\right)S_{2},\\ \frac{dE_{1}}{dt} & = & \beta_{h1}S_{1}W-\left(\delta_{1}+\mu_{h}\right)E_{1},\\ \frac{dE_{2}}{dt} & = & \beta_{h2}S_{2}W-\left(\delta_{2}+\mu_{h}\right)E_{2},\\ \frac{dI}{dt} & = & \delta_{1}E_{1}+\delta_{2}E_{2}+\left(1-p-q\right)\gamma_{2}T-\left(u_{2}\left(t\right)+\gamma_{1}+\mu_{h}\right)I\\ \frac{dT}{dt} & = & u_{2}\left(t\right)I-p\gamma_{2}T-q\gamma_{2}T-\left(1-p-q\right)\gamma_{2}T-\mu_{h}T,\\ \frac{dR_{1}}{dt} & = & \gamma_{1}I+q\gamma_{2}T-\left(\kappa +\mu_{h}\right)R_{1},\\ \frac{dR_{2}}{dt} & = & p\gamma_{2}T-\left(\vartheta +\mu_{h}\right)R_{2},\\ \frac{dV}{dt} & = & \Pi_{v}-\beta_{v}\left(\zeta_{e1}E_{1}+\zeta_{e2}E_{2}+I+\zeta_{t}T+\zeta_{r}R_{1}\right)V-\mu_{v}V,\\ \frac{dW}{dt} & = & \beta_{v}\left(\zeta_{e1}E_{1}+\zeta_{e2}E_{2}+I+\zeta_{t}T+\zeta_{r}R_{1}\right)V-\mu_{v}W, \end{array}$$

with positive initial conditions, and time measured in months. Next, we deduce the related cost which needed to be minimized in the context of malaria control strategy.

1. **Cost to implement pre-erythtocytic vaccine.** The total cost to implement the pre-erythrocytic vaccine is given by
  $$\int_{0}^{t_{f}}\left(\omega_{1}u_{1}^{2}\right)dt,$$
  where $\omega_{1}$ is the weight parameter for pre-erythrocytic parameter and $t_{f}$ is the final time of simulation. The unit of $\omega_{1}$ is $\frac{1}{{\mathrm{month}}^{2}}$. In this article, we consider the non-linearity term on this cost term as authors in [18,26].
2. **Cost to implement transmission-blocking treatment.** Similar to $u_{1}$, we also use a quadratic term for $u_{2}$. Hence, the total cost of transmission blocking is given by
  $$\int_{0}^{t_{f}}\left(\omega_{2}u_{2}^{2}\right)dt,$$
  where $\omega_{2}$ is the weight parameter for transmission blocking. The unit of $\omega_{2}$ is $\frac{1}{{\mathrm{month}}^{2}}$.
3. **Cost related to the high number of infected individuals.** Except the cost related to the implementation of pre-erythrocytic and transmission-blocking, the cost for malaria control strategy also comes from the cost related to the high number of infected individuals who were not treated (*I* and $R_{1}$). This cost is given by
  $$\int_{0}^{t_{f}}\left(\omega_{3}I+\omega_{4}R_{1}\right)dt,$$
  where $\omega_{3}$ and $\omega_{4}$ are the weight parameters for *I* and $R_{1}$, respectively. $\omega_{3}I$ and $\omega_{4}R_{1}$ respectively denote the cost associated with the high number of infected individuals, such as for maintaining health campaigns or any other related cost which comes as a consequence of the high number of infected individuals. The unit of $\omega_{3}$ and $\omega_{4}$ is $\frac{1}{\mathrm{individual}}$.

Based on the above explanation, the cost function for our model is given by:

(2) $$J=\int_{0}^{t_{f}}\left(\omega_{1}u_{1}^{2}+\omega_{2}u_{2}^{2}+\omega_{3}I+\omega_{4}R_{1}\right)dt.$$

There are no exact values of $\omega_{i}$ for i=1,2,3,4. Choosing the values of $\omega_{i}$ should balance the value of each components on J, since the range of $u_{1}$ and $u_{2}$ are very small compared to *I* and $R_{1}$. Hence, we have to choose $\omega_{i}$ so that no component dominates other components in the cost function. For example, in order to balance between $\omega_{1}u_{1}^{2}$ and $\omega_{3}I$, then $\omega_{1}$ and $\omega_{3}$ should satisfy $\frac{\omega_{1}}{\omega_{3}}\approx \frac{I}{u_{1}^{2}}$. To simulate our optimal control problem, it is important that we use parameter values which can present the situation of malaria dynamics in Papua and West papua. Hence, it is important that we estimate our parameter values based on the incidence data in these areas.

### 2.2. Parameter Estimation

In this section, we discuss the incidence data that we use to estimate our parameters and the numerical scheme that has been used. The incidence data for malaria are taken from two provinces in Indonesia that have the highest number of malaria incidence every year, namely Papua and West Papua [44]. According to [32,33], the total number of inhabitants in Papua and West Papua was 3,435,430 and 981,822 in 2020, respectively. In both areas, the majority of malaria cases are caused by *Plasmodium falciparum*, and *Plasmodium vivax*, both of which cause routine health problems and morbidity in these areas [45].

**Figure 2:**
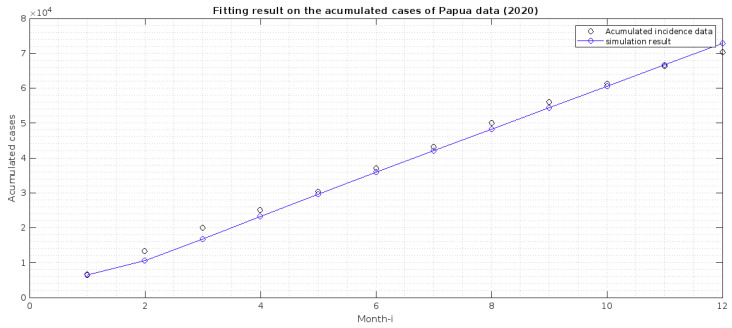
Estimation result on fitting the accumulated cases in Papua with c(t) in Equation (3). The best-fit initial conditions for $S_{1},$ $E_{1},$ I, T, $R_{1},$ $R_{2},$ V,*W* are 3,435,430, 2000, 2000, 2000, 1000, 21, 6,870,860, 3000, respectively.

The incidence data used in this article is the monthly new cases in both provinces, from January to December 2020. The data was collected by personal request from the Directorate of Prevention and Control of Vector and Zoonotic Infectious Diseases, Ministry of Health of the Republic of Indonesia. The data can be seen in Figure 2.

In our model, we do not have a compartment that describes the monthly cases of malaria. Hence, we need to adapt our model to accommodate this. Hence, we transform the monthly cases in both provinces into the accumulated case. Next, we create a new compartment from our model, which describes the accumulated cases. From the transmission diagram given in Figure 1, the total detected cases are coming from the compartment *T*. Hence, the newly detected cases come from the intervention of transmission-blocking from *I* to *T* with a rate of $u_{2}$. Hence, the dynamic of the monthly cases is given by

(3) $$\frac{dc}{dt}=u_{2}I.$$

**Figure 3:**
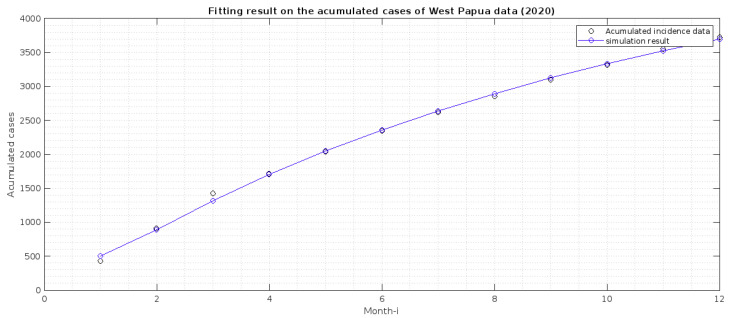
Estimation result of fitting the accumulated cases in West Papua with c(t) in Equation (3). The best-fit initial conditions for $S_{1},$ $E_{1},$ I, T, $R_{1},$ $R_{2},$ V, *W* are 974,300, 400, 400, 51, 21, 125, 1,789,099, 202, respectively.

Solving the above differential equation will give us the accumulated cases of malaria incidence. Our task is to minimize the Euclidean distance between c(t) from our numerical results with the incidence data, using the best-fit parameter of our model. This task can be formulated as follows.

(4) $$\min_{X}\int_{0}^{t_{f}}{\left(c{\left(t\right)}^{\mathrm{simulation}}-c{\left(t\right)}^{\mathrm{data}}\right)}^{2}dt,$$

where *X* is the set of best-fit parameters and initial conditions, while $t_{f}$ is the final time of available data. However, because the use of pre-erythrocytic vaccines had not been implemented in Papua and West Papua in 2020, parameter estimation is performed when the parameters $u_{1},\alpha ,\beta_{h2},\delta_{2},$ and $\zeta_{e2}$ are zero and the $S_{2}$ and $E_{2}$ compartments are zero. The corresponding best-fit parameters are $\kappa ,\vartheta ,\bar{\beta_{h1}},b,\delta_{1},p,q,u_{2},\gamma_{1},\gamma_{2},\bar{\beta_{v}},\zeta_{e1},\zeta_{t},\zeta_{r},$ and all initial conditions of the system (1) (except $S_{2}\left(0\right)$ and $E_{2}\left(0\right)$) and (3). We used a nonlinear optimization toolbox in MATLAB to conduct this parameter estimation, called *fmincon* function. To run the simulation, we need to give an estimation of an interval in which our parameter values must exist. Hence, we use the lower and upper bound of our parameter values, calculated as shown in Table 2. We estimate our parameter values for incidence data in Papua and West Papua, and the result is given in Table 1, while the fitted cumulated cases are in Figure 2 and Figure 3. This finding indicates that malaria incidence will increase in Papua Province, which tends to the malaria-endemic equilibrium point. Meanwhile, malaria incidence in West Papua Province tends to the malaria-free equilibrium point. From our modeling analyses/results, we can deduce the following:

1. Papua is more malaria-endemic than West Papua given the non-controlled basic reproduction number ($R_{0}{|}_{\mathrm{Papua}}=1.75$ and $R_{0}{|}_{\mathrm{West}\;\mathrm{Papua}}=1.53$). See the formula of non-controlled basic reproduction number ($R_{0}$) in (12).
2. Based on the value of *p* and *q*, we conclude that individuals in Papua have a slightly bigger chance of reaching the malaria elimination target if there is a continuous implementation of treatment efforts compared to West Papua.
3. The infection rates from mosquitoes to humans $(\beta_{h1},$ and $\beta_{h2})$ in West Papua is higher than in Papua. Hence, it is important to develop a media campaign that targets reducing the contact between humans and mosquitoes in West Papua than in Papua. Such campaigns may include the use of bed nets or mosquito repellent.

## 3. Dynamical Analysis of the Model

### 3.1. Preliminary Results on the Positiveness and Boundedness of the Solution

The malaria model in system (1) involves human and mosquito populations. Hence, it is necessary to guarantee that all associated variables are positive with nonnegative parameters and initial conditions.

**Theorem** **1.** *Let the initial conditions of all variables in system (1) be nonnegative as follows:*

(5) $$\begin{array}{c} S_{1}\left(0\right)>0,\;\;\;\;\;S_{2}\left(0\right)\geq 0,\;\;\;\;\;E_{1}\left(0\right)\geq 0,\;\;\;\;\;E_{2}\left(0\right)\geq 0,\;\;\;\;\;I\left(0\right)\geq 0,\\ T\left(0\right)\geq 0,\;\;\;\;\;R_{1}\left(0\right)\geq 0,\;\;\;\;\;T_{2}\left(0\right)\geq 0,\;\;\;\;\;V\left(0\right)>0,\;\;\;\;\;W\left(0\right)\geq 0. \end{array}$$

*Then the solution set*

$$\Omega =\left\{S_{1}\left(t\right),S_{2}\left(t\right),E_{1}\left(t\right),E_{2}\left(t\right),I\left(t\right),T\left(t\right),R_{1}\left(t\right),R_{2}\left(t\right),V\left(t\right),W\left(t\right)\right\}$$

*is nonnegative for all t≥0.*

**Proof.**  Let $\beta_{h1}S_{1}W+\left(u_{1}+\mu_{h}\right)S_{1}=G\left(t\right)$. Since the initial conditions of $R_{1},R_{2},$ and $S_{2}$ are nonnegative, then the first equation in system (1) satisfies:

$$\begin{array}{ccc} \frac{dS_{1}\left(t\right)}{dt} & = & \Pi_{h}-G\left(t\right)S_{1}\left(t\right)+\kappa R_{1}+\vartheta R_{2}+\alpha S_{2}\\ & \geq & \Pi_{h}-G\left(t\right)S_{1}\left(t\right). \end{array}$$

Hence, we have:

$$\frac{dS_{1}\left(t\right)}{dt}+G\left(t\right)S_{1}\left(t\right)\geq \Pi_{h}.$$

Choosing the integrating factor as $\exp\left(\int_{0}^{t}G\left(\tau \right)d\tau .\right)$ gives us:

$$\begin{array}{ccc} \frac{dS_{1}\left(t\right)}{dt}\exp\left(\int_{0}^{t}G\left(\tau \right)d\tau \right)+G\left(t\right)S_{1}\left(t\right)\exp\left(\int_{0}^{t}G\left(\tau \right)d\tau \right) & \geq & \Pi_{h}\exp\left(\int_{0}^{t}G\left(\tau \right)d\tau \right)\\ \frac{d}{dt}\left[S_{1}\left(t\right)\exp\left(\int_{0}^{t}G\left(\tau \right)d\tau \right)\right] & \geq & \Pi_{h}\exp\left(\int_{0}^{t}G\left(\tau \right)d\tau \right). \end{array}$$

Integrating both sides yields

(6) $$S_{1}\left(t\right)\exp\left(\int_{0}^{t}G\left(\tau \right)d\tau \right)-S_{1}\left(0\right)\geq \Pi_{h}\int_{0}^{t}\exp\left(\int_{0}^{\tau }G\left(u\right)du\right)dt.$$

Therefore,

(7) $$S_{1}\left(t\right)\geq S_{1}\left(0\right)\exp\left(-\int_{0}^{\tau }G\left(u\right)du\right)+\Pi_{h}\left(\int_{0}^{t}\exp\left(\int_{0}^{\tau }G\left(u\right)du\right)dt\right)\exp\left(-\int_{0}^{\tau }G\left(u\right)du\right)\geq 0.$$

Therefore, we can see that if $S_{1}\left(0\right)>0$, then we have that $S_{1}\left(t\right)\geq 0$ for all t>0. A similar approach can be used to show that $S_{2}\left(t\right),E_{1}1\left(t\right),E_{2}\left(t\right),I\left(t\right),R_{1}\left(t\right),R_{2}\left(t\right),V\left(t\right),$ and W(t) are nonnegative for all t≥0. This completes the proof. □

It can be shown that our model in system (1) is well-defined biologically in the feasible region below.

(8) $$\Omega =\left\{\left(X\left(t\right),Y\left(t\right)\right)\in R_{+}^{8}\times R_{+}^{2}|0\leq N\left(t\right)\leq \frac{\Pi_{h}}{\mu_{h}},0\leq V\left(t\right)+W\left(t\right)\leq \frac{\Pi_{v}}{\mu_{v}}\right\},$$

where $X\left(t\right)=\{S_{1}\left(t\right),S_{2}\left(t\right),E_{1}\left(t\right),E_{2}\left(t\right),I\left(t\right),T\left(t\right),R_{1}\left(t\right),R_{2}\left(t\right)\}$ and Y(t)={V(t),W(t)}.

### 3.2. The Malaria-Free Equilibrium and the Controlled Reproduction Number

The first equilibrium point of malaria model in (1) is the malaria-free equilibrium, which is denoted by $E^{*}$, and given by

(9) $$\begin{array}{cc} E^{*} & =(S_{1}^{0},S_{2}^{0},E_{1}^{0},E_{2}^{0},I^{0},T^{0},R_{1}^{0},R_{2}^{0},V^{0},W^{0})\\ & =\left(\frac{\left(\alpha +\mu_{h}\right)\Pi_{h}}{\mu_{h}\left(\alpha +\mu_{h}+u_{1}\right)},\frac{u_{1}\Pi_{h}}{\mu_{h}\left(\alpha +\mu_{h}+u_{1}\right)},0,0,0,0,0,0,\frac{\Pi_{v}}{\mu_{v}},0\right). \end{array}$$

It can be seen that $E^{*}$ presents a condition where all infections have disappeared from both populations. In this condition, we also can see that the ratio of susceptible human beings with and without pre-erythrocytic vaccine is given by

(10) $$\begin{array}{c} \frac{S_{1}^{0}}{S_{2}^{0}}=\frac{\frac{\left(\alpha +\mu_{h}\right)\Pi_{h}}{\mu_{h}\left(\alpha +\mu_{h}+u_{1}\right)}}{\frac{u_{1}\Pi_{h}}{\mu_{h}\left(\alpha +\mu_{h}+u_{1}\right)}}=\frac{\left(\alpha +\mu_{h}\right)\Pi_{h}}{u_{1}\Pi_{h}}=\frac{\alpha +\mu_{h}}{u_{1}}. \end{array}$$

The purpose is, of course, to reduce this ratio, which is equivalent to increasing the number of individuals in $S_{2}$. It can be seen that this ratio is only affected by three parameters, namely $\mu_{h},\alpha ,$ and $u_{1}$. Of these three parameters, only the latter two are controllable. Therefore, we can see that reducing this ratio can be done by increasing the rate of vaccination, and the vaccine’s duration could be prolonged. Hence, the higher the quality of the vaccine (i.e., its duration), the better.

We will now calculate the basic and controlled reproduction number of our model, which is denoted by $R_{0}$ and $R_{C}$, respectively. The basic reproduction number defines the expected average number of secondary malaria cases caused by a single malaria-infected case during its infection period in a completely susceptible population. This threshold holds a vital role in many malaria models [48]. From its definition, $R_{0}$ helps us to understand the qualitative behavior of our model and whether the disease may persist or exist. Mostly, they found that malaria will disappear from the population if $R_{0}<1$, and exist if $R_{0}>1$. In several cases, mainly when backward bifurcation phenomena occur in their model [18,49,50,51], it is still possible to find malaria even though $R_{C}$ is already less than one.

When no control intervention is given to our model, then system (1) reduced into:

(11a) $$\begin{array}{cc} \frac{dS_{1}}{dt} & =\Pi_{h}+\kappa R_{1}-\beta_{h1}S_{1}W-\mu_{h}S_{1}, \end{array}$$

(11b) $$\begin{array}{cc} \frac{dE_{1}}{dt} & =\beta_{h1}S_{1}W-\left(\delta_{1}+\mu_{h}\right)E_{1}, \end{array}$$

(11c) $$\begin{array}{cc} \frac{dI}{dt} & =\delta_{1}E_{1}-\left(\gamma_{1}+\mu_{h}\right)I, \end{array}$$

(11d) $$\begin{array}{cc} \frac{dR_{1}}{dt} & =\gamma_{1}I-\left(\kappa +\mu_{h}\right)R_{1}, \end{array}$$

(11e) $$\begin{array}{cc} \frac{dV}{dt} & =\Pi_{v}-\beta_{v}\left(\zeta_{e1}E_{1}+I+\zeta_{r}R_{1}\right)V-\mu_{v}V, \end{array}$$

(11f) $$\begin{array}{cc} \frac{dW}{dt} & =\beta_{v}\left(\zeta_{e1}E_{1}+I+\zeta_{r}R_{1}\right)V-\mu_{v}W. \end{array}$$

To find the basic reproduction number of the basic model (Section 3.2), we use the well known next-generation matrix approach [52]. The respected basic reproduction number for the basic model (11) is given as follows.

(12) $$R_{0}=\sqrt{\left(\frac{\Pi_{h}\beta_{h1}}{\mu_{h}\mu_{v}}\right)\times \left(\frac{\beta_{v}\zeta_{e1}\pi_{v}}{\mu_{v}\left(\delta_{1}+\mu_{h}\right)}+\frac{\beta_{v}\pi_{v}\delta_{1}}{\mu_{v}\left(\delta_{1}+\mu_{h}\right)\left(\gamma_{1}+\mu_{h}\right)}+\frac{\beta_{v}\zeta_{r}\Pi_{v}\delta_{1}\gamma_{1}}{\mu_{v}\left(\delta_{1}+\mu_{h}\right)\left(\gamma_{1}+\mu_{h}\right)\left(\kappa +\mu_{h}\right)}\right)}.$$

With a similar approach, we can calculate the controlled reproduction number of our malaria problem. Implementing the next-generation matrix method into system (1), the controlled reproduction number is given by:

(13) $$R_{C}=\sqrt{fa+gb+hc+id+je},$$

where

(14a) $$\begin{array}{cc} fa & =\underset{R_{0v}^{E_{1}}}{\underbrace{\frac{\Pi_{v}}{\mu_{v}}\cdot \frac{\beta_{v}\zeta_{e1}}{\mu_{v}}}}\cdot \underset{R_{0h1}^{E_{1}}}{\underbrace{\frac{\Pi_{h}}{\mu_{h}}\cdot \frac{\alpha +\mu_{h}}{\alpha +\mu_{h}+u_{1}}\cdot \frac{\beta_{h1}}{\delta_{1}+\mu_{h}}}}, \end{array}$$

(14b) $$\begin{array}{cc} gb & =\underset{R_{0v}^{E_{2}}}{\underbrace{\frac{\Pi_{v}}{\mu_{v}}\cdot \frac{\beta_{v}\zeta_{e2}}{\mu_{v}}}}\cdot \underset{R_{0h2}^{E_{2}}}{\underbrace{\frac{\Pi_{h}}{\mu_{h}}\cdot \frac{u_{1}}{\alpha +\mu_{h}+u_{1}}\cdot \frac{\beta_{h2}}{\delta_{2}+\mu_{h}}}}, \end{array}$$

(14c) $$\begin{array}{cc} hc & =\underset{R_{0v}^{I}}{\underbrace{\frac{\Pi_{v}}{\mu_{v}}\cdot \frac{\beta_{v}}{\mu_{v}}}}\cdot \underset{R_{0h}^{I}}{\underbrace{\frac{\Pi_{h}}{\mu_{h}}K}}, \end{array}$$

(14d) $$\begin{array}{cc} id & =\underset{R_{0v}^{T}}{\underbrace{\frac{\Pi_{v}}{\mu_{v}}\cdot \frac{\beta_{v}\zeta_{t}}{\mu_{v}}}}\cdot \underset{R_{0h}^{T}}{\underbrace{\frac{\Pi_{h}}{\mu_{h}}\left(\frac{\alpha +\mu_{h}}{\alpha +\mu_{h}+u_{1}}\cdot \frac{\beta_{h1}}{\delta_{1}+\mu_{h}}\cdot \frac{\delta_{1}u_{2}}{L}+\frac{u_{1}}{\alpha +\mu_{h}+u_{1}}\cdot \frac{\beta_{h2}}{\delta_{2}+\mu_{h}}\cdot \frac{\delta_{2}u_{2}}{L}\right)}}, \end{array}$$

(14e) $$\begin{array}{cc} je & =\underset{R_{0v}^{R_{1}}}{\underbrace{\frac{\Pi_{v}}{\mu_{v}}\cdot \frac{\beta_{v}\zeta_{r}}{\mu_{v}}}}\cdot \underset{R_{0h}^{R_{1}}}{\underbrace{\frac{\Pi_{h}}{\mu_{h}}M}}, \end{array}$$

and

$$\begin{array}{cc} K & =\frac{\alpha +\mu_{h}}{\alpha +\mu_{h}+u_{1}}\cdot \frac{\beta_{h1}}{\delta_{1}+\mu_{h}}\cdot \frac{\delta_{1}\left(\gamma_{2}+\mu_{h}\right)}{L}+\frac{u_{1}}{\alpha +\mu_{h}+u_{1}}\cdot \frac{\beta_{h2}}{\delta_{2}+\mu_{h}}\cdot \frac{\delta_{2}\left(\gamma_{2}+\mu_{h}\right)}{L},\\ M & =\frac{\alpha +\mu_{h}}{\alpha +\mu_{h}+u_{1}}\cdot \frac{\beta_{h1}}{\delta_{1}+\mu_{h}}\cdot \frac{\delta_{1}}{\kappa +\mu_{h}}\cdot \frac{u_{2}\gamma_{2}q+\gamma_{2}\gamma_{1}+\mu_{h}\gamma_{1}}{L}\cdots \\ \; & +\frac{u_{1}}{\alpha +\mu_{h}+u_{1}}\cdot \frac{\beta_{h2}}{\delta_{2}+\mu_{h}}\cdot \frac{\delta_{2}}{\kappa +\mu_{h}}\cdot \frac{u_{2}\gamma_{2}q+\gamma_{2}\gamma_{1}+\mu_{h}\gamma_{1}}{L},\\ L & =p\gamma_{2}u_{2}+q\gamma_{2}u_{2}+\gamma_{1}\gamma_{2}+\gamma_{1}\mu_{h}+\gamma_{2}\mu_{h}+{\mu_{h}}^{2}+\mu_{h}u_{2}. \end{array}$$

As we can see from the expression on (13), $R_{0}$ is the combination of many paths of infection on our model. The explanation is given as follows.

1. $R_{0v}^{E_{1}}$ shows the path of transmission for a new case in mosquito due to a bite from susceptible mosquitoes to an exposed human in compartment $E_{1}$.
2. $R_{0h1}^{E_{1}}$ shows the transmission path which gives a new infection in humans without pre-erythrocytic vaccine $\left(E_{1}\right)$ due to a bite from infected mosquitoes.
3. $R_{0v}^{E_{2}}$ presents the transmission path for a new infection in the mosquito population after they bite exposed humans in $E_{2}$.
4. $R_{0h2}^{E_{2}}$ presents the transmission path for a new infection in vaccinated human $E_{2}$ due to a bite from infected mosquitoes.
5. $R_{0v}^{I}$ shows a transmission for a new infection in the mosquitoes population after biting an infected individual in *I*.
6. $R_{0h}^{I}$ presents a transmission path for a new cases in *I* due to progression of $E_{1}$ and $E_{2}$.
7. $R_{0v}^{T}$ presents a transmission path for a new case in the mosquitoes population after biting an infected individual who is undergoing treatment (T).
8. $R_{0h}^{T}$ presents a transmission path for a new case in treated human population (T) due to the treatment rate from *I*.
9. $R_{0v}^{R_{1}}$ presents a transmission path for a new case in the mosquito population after biting individuals in $R_{1}$.
10. $R_{0h}^{R_{1}}$ presents a transmission path for new cases in the compartment of humans who partially succeed in conducting transmission-blocking treatment.

### 3.3. The Malaria-Endemic Equilibrium

The malaria-endemic equilibrium point of system (1) indicates the condition when malaria persists in the human and mosquito populations. The malaria-endemic equilibrium of system (1) will be determined when the number of infected individuals is not equal to zero. However, due to the complexity of the model, we can not explicitly show the form of the endemic equilibrium point as a function of other parameters. Hence, we write our endemic equilibrium point as a function of dependent variables *I* and *W*. The idea is as follows. We set the right hand side of system (1) equal to zero, and solve them backwardly with respect to each variables until we only have *I* and *W* left. This process will give us two different polynomial as a function of *I* and *W*. Hence, the malaria-endemic equilibrium of system (1) is given by:

(15) $$\begin{array}{c} E^{†}=\left(S_{1}^{*},S_{2}^{*},E_{1}^{*},E_{2}^{*},I^{*},T^{*},R_{1}^{*},R_{2}^{*},V^{*},W^{*}\right), \end{array}$$

where

(16a) $$\begin{array}{cc} S_{1}^{*} & =\frac{\left(W^{*}\beta_{h2}+\alpha +\mu_{h}\right)I^{*}\;\left(\delta_{2}+\mu_{h}\right)\left(\delta_{1}+\mu_{h}\right)a}{W^{*}}, \end{array}$$

(16b) $$\begin{array}{cc} S_{2}^{*} & =\frac{u_{1}I^{*}\;\left(\delta_{2}+\mu_{h}\right)\left(\delta_{1}+\mu_{h}\right)a}{W^{*}}, \end{array}$$

(16c) $$\begin{array}{cc} E_{1}^{*} & =\left(W^{*}\beta_{h2}+\alpha +\mu_{h}\right)I^{*}\;\left(\delta_{2}+\mu_{h}\right)\beta_{h1}a, \end{array}$$

(16d) $$\begin{array}{cc} E_{2}^{*} & =u_{1}I^{*}\;\beta_{h2}\left(\delta_{1}+\mu_{h}\right)a, \end{array}$$

(16e) $$\begin{array}{cc} T^{*} & =\frac{u_{2}I^{*}}{\gamma_{2}+\mu_{h}}, \end{array}$$

(16f) $$\begin{array}{cc} R_{1}^{*} & =\frac{\left(\left(qu_{2}+\gamma_{1}\right)\gamma_{2}+\gamma_{1}\mu_{h}\right)I^{*}}{\left(\gamma_{2}+\mu_{h}\right)\left(\kappa +\mu_{h}\right)}, \end{array}$$

(16g) $$\begin{array}{cc} R_{2}^{*} & =\frac{I^{*}\;p\gamma_{2}u_{2}}{\left(\gamma_{2}+\mu_{h}\right)\left(\mu_{h}+\vartheta \right)}, \end{array}$$

(16h) $$\begin{array}{cc} V^{*} & =\frac{\mu_{v}W^{*}}{\beta_{v}\left(T^{*}\zeta_{t}+E_{1}^{*}\zeta_{e1}+E_{2}^{*}\zeta_{e2}+R_{1}^{*}\zeta_{r}+I^{*}\right)}, \end{array}$$

with

$$\begin{array}{cc} a= & \frac{{\mu_{h}}^{2}+\left(\gamma_{1}+\gamma_{2}+u_{2}\right)\mu_{h}+\gamma_{2}\left(\left(p+q\right)u_{2}+\gamma_{1}\right)}{\gamma_{2}+\mu_{h}}\\ & \times \frac{1}{\beta_{h1}\delta_{1}{\mu_{h}}^{2}+\left(\delta_{1}\left(W^{*}\beta_{h2}+\alpha +\delta_{2}\right)\beta_{h1}+u_{1}\delta_{2}\beta_{h2}\right)\mu_{h}+\delta_{2}\left(\left(W^{*}\beta_{h2}+\alpha \right)\beta_{h1}+\beta_{h2}u_{1}\right)\delta_{1}}, \end{array}$$

while $I^{*}$ and $W^{*}$ are taken from the positive intersection of the following polynomials:

(17) $$\begin{array}{c} G_{1}=a_{2}\left(I\right)W^{2}+a_{1}\left(I\right)W+a_{0}\left(I\right)=0,\\ G_{2}=b_{1}\left(W\right)I+b_{0}\left(W\right)=0, \end{array}$$

where $a_{i}\left(I\right),b_{i}\left(W\right)$ for i=0,1,2 has a complex form to be shown. We leave the existence analysis on this malaria-endemic equilibrium as an open problem to an interested reader.

We show the existence of this malaria-endemic equilibrium numerically with the following scenario. To conduct this numerical experiment, we use the following parameter values:

Using the above parameter values, we have the basic reproduction number of system (1) is 1.002169498, which is larger than one. Substituting the parameter values in Table 3 into $G_{1}$ and $G_{2}$ yield:

(18) $$\begin{array}{cc} G_{1} & =\left(2.81\times 10^{-19}I+2.4\times 10^{-11}\right)W^{2}+\left(-1.89\times 10^{-12}I+3.62\times 10^{-6}\right)W-2.91\times 10^{-7}I=0,\\ G_{2} & =6.14\times 10^{-15}\left(W+150489.34\right)\left(W+34834.79\right)I-2.67\times 10^{-9}\left(W+150616.39\right)W=0. \end{array}$$

**Figure 4:**
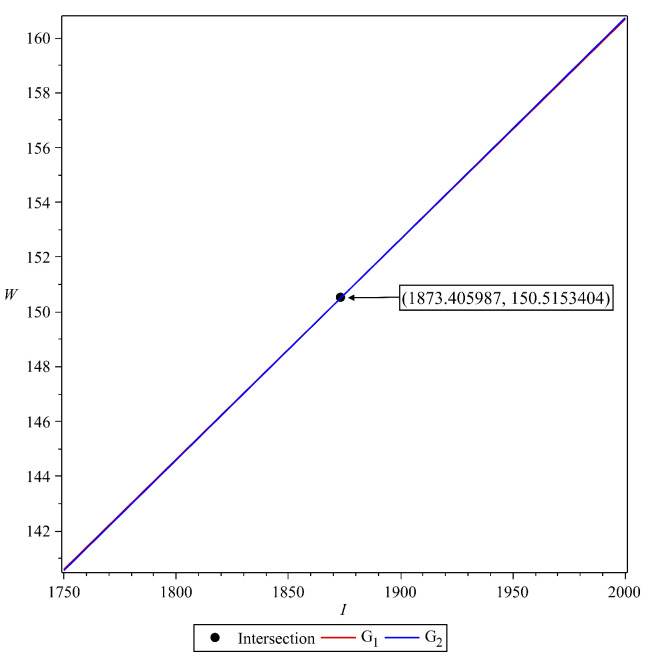
Illustration for the existence of malaria-endemic equilibrium point.

Next, we plot $G_{1}$ and $G_{2}$ in I−W plane. The result is given in Figure 4.

Since we have an intersection between $G_{1}$ and $G_{2}$ in the first quadrant of I−W plane, then we have the malaria-endemic equilibrium point, which is given by:

(19) $$\begin{array}{cc} E^{†}= & \left(S_{1}^{*},S_{2}^{*},E_{1}^{*},E_{2}^{*},I^{*},T^{*},R_{1}^{*},R_{2}^{*},V^{*},W^{*}\right)\\ = & \left(3400396,20241,200,2,1874,862,1960,9898,6870710,151\right). \end{array}$$

## 4. Sensitivity Analysis

### 4.1. Global Sensitivity Analysis on the Basic Reproduction Number

**Figure 5:**
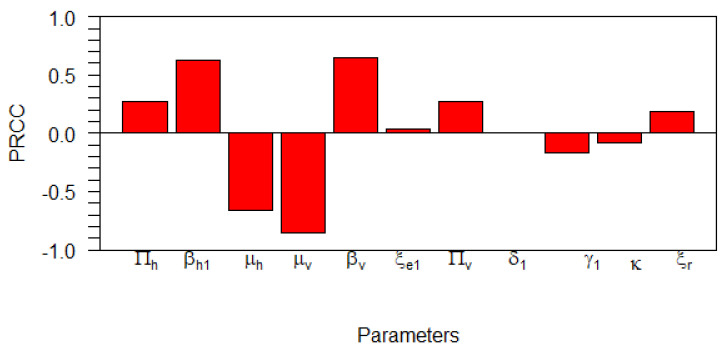
Tornado plot showing the PRCC values for the model parameters in $R_{0}$.

In this subsection, we carry out a global uncertainty analysis of basic reproduction number for the malaria model without control. As we have already shown in the previous section, the basic reproduction number could determine whether malaria will persist or become extinct in the population. Hence, it is essential to analyze the sensitivity of the basic reproduction number with respect to the model parameters. Global sensitivity analysis is used to study the relative changes in epidemic model parameters output, giving input/altered model parameters for the dynamical systems under investigation. Thus, we enabled modelers to identify key model variables that require controlling for the particular disease. To carry out the simulation, we used global sensitivity analysis, which combines the Latin-hypercube sampling and Partial Rank Cross Correlation (PRCC) technique as given in [53] with similar analysis in [54]. Using R-software, we performed 1000 simulations per run, and examined and evaluated the PRCC of the model parameters concerning in $R_{0}.$ The PRCC indicates the degree of monotonicity between the model’s parameters in the $R_{0}$. Thus, juxtaposing the values of PRCC gives an apparent effect and contribution of each model parameter on $R_{0}$ in our malaria model. The results from the numerical simulations are given in Figure 5 and Table 4. The sensitivity of the parameters to the basic reproduction number (12) is proportional to the absolute value of PRCC. As we can see in Table 4, we observe $\Pi_{h},\beta_{h1},\mu_{h},\mu_{v},\beta_{v},\Pi_{v},\gamma_{1},$ and $\xi_{r}$. From these parameters, we can see that $\beta_{h1},\beta_{v},$ and $\mu_{v}$ are the most significant parameters that can be controlled. Increasing $\mu_{v}$ and reducing $\beta_{h1}$ and $\beta_{v}$ will reduce $R_{0}$. It can also been seen in Table 4 that all the parameters have *p*-values <0.05 and thus are said to be significant besides $\xi_{e1},\;\kappa ,$ and $\delta_{1}.$ Therefore, it is essential to introduce intervention to reduce the infection rate with vaccine in susceptible populations, or increasing the mosquito death rate with fumigation.

### 4.2. Local Sensitivity Analysis on the Model Variables

The sensitivity methods can be used on infectious disease models to determine which variable or parameter is sensitive to a specific condition. In our case, identifying the key critical parameters of system (1) is an effective way to study the qualitative behaviour of the parameters which govern the model. In our proposed model (1), we have 10 compartments $x_{i}$ for i=1,2,...,10 and 22 parameters $k_{j}$ for j=1,2,...,22. Then, the local sensitivities can be calculated using three different techniques: non-normalisations, half-normalizations, and full-normalizations. Firstly, the equation of non-normalization sensitivities is given by

(20) $$\begin{array}{c} S_{k_{j}}^{x_{i}}=\frac{\partial x_{i}}{\partial k_{j}}, \end{array}$$

where $S_{k_{j}}^{x_{i}}$ is measured as sensitivity coefficients of each $x_{i}$ with respect to each parameter $k_{j}$. Then, the formula of half–normalization sensitivities is defined below:

(21) $$\begin{array}{c} S_{k_{j}}^{x_{i}}=\left(\frac{1}{x_{i}}\right)\left(\frac{\partial x_{i}}{\partial k_{j}}\right). \end{array}$$

Finally, the equation of full-normalization sensitivities is defined by

(22) $$\begin{array}{c} S_{k_{j}}^{x_{i}}=\left(\frac{k_{j}}{x_{i}}\right)\left(\frac{\partial x_{i}}{\partial k_{j}}\right). \end{array}$$

**Figure 6:**
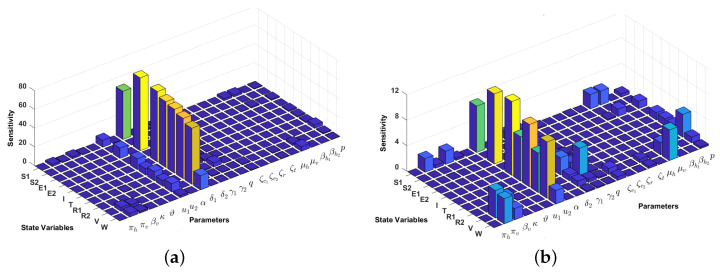
Local sensitivity analysis with full–normalization technique of all variables with respect to all parameters (**a**) and without $\delta_{1}$ (**b**). We use incidence data from Papua and the following initial conditions: $S_{1}\left(0\right)=3,208,839,$ $S_{2}\left(0\right)=0,\;E_{1}\left(0\right)=2000,\;E_{2}\left(0\right)=0,\;I\left(0\right)=2000,\;T\left(0\right)=1000,\;R_{1}\left(0\right)=1000,\;R_{2}\left(0\right)=317,\;V\left(0\right)=6,664,102,\;W\left(0\right)=2781$ to run the simulation.

**Figure 7:**
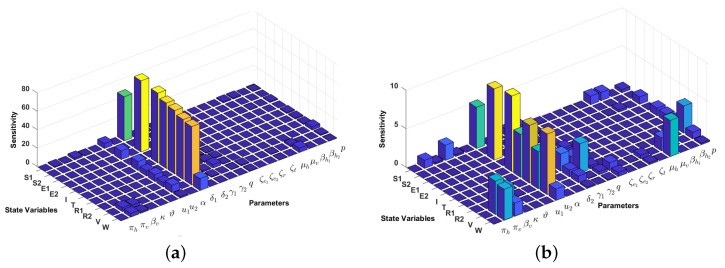
Local sensitivity analysis with full–normalization technique of all variables with respect to all parameters (**a**) and without $\delta_{1}$ (**b**). We use incidence data from West-Papua and the following initial conditions: $S_{1}\left(0\right)=918,167,\;S_{2}\left(0\right)=0,\;E_{1}\left(0\right)=399,\;E_{2}\left(0\right)=0,\;I\left(0\right)=242,\;T\left(0\right)=51,\;R_{1}\left(0\right)=22,\;R_{2}\left(0\right)=88,\;V\left(0\right)=1,915,967,\;W\left(0\right)=641$ to run the simulation.

In this article, we only perform a full-normalization sensitivity analysis on our proposed model in (1) for best-fit parameter of Papua and West-Papua incidence data. The results are given in Figure 6 and Figure 7. We can see from Figure 6 and Figure 7 that the progression rate from $E_{1}$ to *I* ($\delta_{1}$) is the most influential parameter for all compartments, except $S_{2}$ and $E_{2}$. The second most influential parameter is the transmission-blocking parameter $\left(u_{2}\right)$. In contrast to $u_{2}$, we can see that the impact of pre-erythrocytic $\left(u_{1}\right)$ is not as sensitive as $u_{2}$. This result is similar to our previous sensitivity analysis on $R_{0}$.

## 5. Existence of Solution and Characterization of the Optimal Control Problem

We consider our optimal control problem on determining $S_{1}^{*},$ $S_{2}^{*},$ $E_{1}^{*},$ $E_{2}^{*},$ $I^{*},$ $T^{*},$ $R_{1}^{*},$ $R_{2}^{*},$ $V^{*},$ $W^{*}$, associated with our admissible control parameter $(u_{1}^{*},u_{2}^{*})\in \Omega$ on the time interval [0,T], which satisfies our malaria model in (1), non-negative initial conditions $S_{1}\left(0\right),$ $S_{2}\left(0\right),$ $E_{1}\left(0\right),$ $E_{2}\left(0\right),$ I(0), T(0), $R_{1}\left(0\right),$ $R_{2}\left(0\right),$ V(0), and W(0) in order to minimize the cost function in (2), i.e.,

(23) $$J\left(u_{1}^{*},u_{2}^{*}\right)=\min_{\Omega }J\left(u_{1},u_{2}\right).$$

The existence of our optimal control solutions $\left(u_{1}^{*},u_{2}^{*}\right)$ comes from the convexity of the integrand of the cost function in (2) with respect to the controls and the regularity of the malaria model in (1) (See [55,56] for the existence results of optimal solutions).

### 5.1. Existence of the Optimal Solution

In this subsection we state and prove the existence of solutions for the optimal control ODE system given in Equation (1), before proving the existence of solutions for the optimal control problem. Firstly, it is necessary to establish the boundedness of our malaria model over the modeling time horizon. Now, let ${\hat{S}}_{1},{\hat{S}}_{2},{\hat{E}}_{1},{\hat{E}}_{2},\hat{I},\hat{T},{\hat{R}}_{1},{\hat{R}}_{2},\hat{V}$, and $\hat{W}$ denote the super-solutions with respect to each state variable in Equation (1). Therefore, we obtain

(24a) $$\begin{array}{cc} \frac{d{\hat{S}}_{1}}{dt} & =\Pi_{h}, \end{array}$$

(24b) $$\begin{array}{cc} \frac{d{\hat{S}}_{2}}{dt} & ={\hat{S}}_{1}, \end{array}$$

(24c) $$\begin{array}{cc} \frac{d{\hat{E}}_{1}}{dt} & =1, \end{array}$$

(24d) $$\begin{array}{cc} \frac{d{\hat{E}}_{2}}{dt} & =1, \end{array}$$

(24e) $$\begin{array}{cc} \frac{d\hat{I}}{dt} & =\delta_{1}{\hat{E}}_{1}+\delta_{2}{\hat{E}}_{2}, \end{array}$$

(24f) $$\begin{array}{cc} \frac{d\hat{T}}{dt} & =\hat{I}, \end{array}$$

(24g) $$\begin{array}{cc} \frac{d{\hat{R}}_{1}}{dt} & =\gamma_{1}\hat{I}+q\gamma_{2}\hat{T}, \end{array}$$

(24h) $$\begin{array}{cc} \frac{d{\hat{R}}_{2}}{dt} & =p\gamma_{2}\hat{T}, \end{array}$$

(24i) $$\begin{array}{cc} \frac{d\hat{V}}{dt} & =1, \end{array}$$

(24j) $$\begin{array}{cc} \frac{d\hat{W}}{dt} & =\beta_{v}\left(\zeta_{e1}{\hat{E}}_{1}+\zeta_{e2}{\hat{E}}_{2}+\hat{I}+\zeta_{t}\hat{T}+\zeta_{r}{\hat{R}}_{1}\right). \end{array}$$

Writing system (24) in a vector notation format, we get

$${\bar{X}}^{\prime }=M_{1}\bar{X}+M_{2},$$

where $\bar{X}=\left(\begin{array}{c} {\hat{S}}_{1}\\ {\hat{S}}_{2}\\ {\hat{E}}_{1}\\ {\hat{E}}_{2}\\ \hat{I}\\ \hat{T}\\ {\hat{R}}_{1}\\ {\hat{R}}_{2}\\ \hat{V}\\ \hat{W} \end{array}\right)$, $M_{1}\left(\begin{array}{cccccccccc} 0 & 0 & 0 & 0 & 0 & 0 & 0 & 0 & 0 & 0\\ 1 & 0 & 0 & 0 & 0 & 0 & 0 & 0 & 0 & 0\\ 0 & 0 & 0 & 0 & 0 & 0 & 0 & 0 & 0 & 0\\ 0 & 0 & 0 & 0 & 0 & 0 & 0 & 0 & 0 & 0\\ 0 & 0 & 0 & 0 & \delta_{1} & \delta_{2} & 0 & 0 & 0 & 0\\ 0 & 0 & 0 & 0 & 0 & 1 & 0 & 0 & 0 & 0\\ 0 & 0 & 0 & 0 & q\gamma_{2} & \gamma_{1} & 0 & 0 & 0 & 0\\ 0 & 0 & 0 & 0 & p\gamma_{2} & \gamma_{1} & 0 & 0 & 0 & 0\\ 0 & 0 & 0 & 0 & 0 & 0 & 0 & 0 & 0 & 0\\ 0 & 0 & \beta_{v}\zeta_{e1} & \beta_{v}\zeta_{e2} & 1 & \beta_{v}\zeta_{t} & \beta_{v}\zeta_{r} & 0 & 0 & 0 \end{array}\right)$ and

$M_{2}=\left(\begin{array}{c} \Pi_{h}\\ 0\\ 1\\ 1\\ 0\\ 0\\ 0\\ 0\\ 1\\ 0 \end{array}\right).$

From the above matrices, it can be deduced that our model is linear and satisfies the properties of being semi-positive definite on Ω. Then, it follows that the super-solutions of the system (1) are bounded.

Applying Theorem 4.1 on pages 68–69 of Fleming and Rishel [56], we then show the existence of the optimal control model. Supposed

$$H=\left\{u_{k}\left(t\right):0\leq u_{k}\left(t\right)\leq 1\right\},\;\;\forall t\in \left[0,t_{f}\right]\;\;for\;\;k=1,2.$$

**Theorem** **2.** *Given the objective functional*

(25) $$J=\int_{0}^{t_{f}}\left(\omega_{1}u_{1}^{2}+\omega_{2}u_{2}^{2}+\omega_{3}I+\omega_{4}R_{1}\right)dt,$$

which has associated admissible control while subject to the model initial conditions given in Theorem 1. There exists an optimal control pair $u_{1}^{*},u_{2}^{*}$ which minimizes the functional

(26) $$J\left(u_{1}^{*},u_{2}^{*}\right)=\min_{\Omega }J\left(u_{1},u_{2}\right).$$

*Therefore, the conditions enumerated below should be satisfied by our model.*

*1.*  *The admissible control parameters and each model state variable are non-empty.*
*2.*  *Control H is convex and bounded.*
*3.*  *Right hand side of our system is continuous and bounded above by a linear function in the state variables and the control parameters.*
*4.*  *The integrand of $J(u_{1}\left(t\right),u_{2}\left(t\right))$ is concave on H.*
*5.*  *There exists positive constants $b_{1},b_{2}>0$ and τ>1 such that $J(u_{k}\left(t\right))$ satisfies*
$$J\left(u_{1}\left(t\right),u_{2}\left(t\right)\right)\leq b_{1}+b_{2}{\left(|u_{1}{|}^{2}+|u_{2}{|}^{2}\right)}^{\frac{\tau }{2}}.$$

**Proof.**  The proof follows for the stated conditions as follows:

1. Applying the methodology of Theorem 9.2.1 on page 182 of [57], the first criteria is fulfilled as the solutions of our model system exist and are bounded in ω as also shown by the existence of the super-solutions.
2. Using the definition of H, we have that set as bounded and closed.
3. Considering our model in Equation (1) and the cost function (2) is linear in $u_{1}$ and $u_{2}.$ with the aid of the result of the theorem as in [57], we can then deduce that the right-hand side of our system is continuous and bounded.
4. Suppose the objective function
  $$L=\omega_{1}u_{1}^{2}+\omega_{2}u_{2}^{2}+\omega_{3}I+\omega_{4}R_{1}$$
  and we set (p,q)∈Θ,forp,qand0≤t≤1. Then L(tp+(1−t)p)≥t(p)+(1−t)Lp. Additionally, let $\left(p,q\right)\in \Theta^{2}$ for 0≤t≤1, then we have that $p=u_{11},\;p=u_{12}$ and $(tp+\left(1-t\right)q=tu_{11}+\left(1-t\right)u_{12}).$ Thus,
  (27a) $$\begin{array}{cc} L(tp+(1-t)q) & =\omega_{3}I+\omega_{4}R_{1}+\omega_{1}{\left(tu_{11}+\left(1-t\right)u_{12}\right)}^{2}+\omega_{2}{\left(tu_{21}+\left(1-t\right)u_{22}\right)}^{2} \end{array}$$
  (27b) $$\begin{array}{cc} tL(p)+(1-t)L(q) & =\omega_{3}I+\omega_{4}R_{1}+\omega_{1}\left(tu_{11}^{2}+\left(1-t\right)u_{12}^{2}\right)+\omega_{2}\left(tu_{21}^{2}+\left(1-t\right)u_{22}^{2}\right). \end{array}$$
  From (2), we observe that
  $${\left(tu_{11}+\left(1-t\right)u_{12}\right)}^{2}\geq \left(tu_{11}^{2}+\left(1-t\right)u_{12}^{2}\right),$$
  $${\left(tu_{21}+\left(1-t\right)u_{22}\right)}^{2}\geq \left(tu_{21}^{2}+\left(1-t\right)u_{22}^{2}\right).$$
  Following this observation, we can then generalize that given any $\left(p,q\right)\in \theta^{2}$ for 0≤t≤1 we obtain L(tp+(1−t)q)≥tL(p)+(1−t)L(q). Hence, the integrand of J is concave.
5. Following the fact that the model state variable in the objective function, that is, *I* and $R_{1}$ are bounded as shown in 3. Above, there exits positive constants $\varphi_{1},\varphi_{2}>0$ such that the sum of $\left(I+R_{1}\right)\leq \varphi_{2}.$ If we set $\varphi_{2}=\max_{n}=3,4\cdots ,$ we have that
  $$L\left(I,R_{1},u_{1},u_{2}\right)\leq \eta_{1}+\eta_{2}+\eta_{3}{\left(|u_{1}^{2}\left|+\right|u_{2}^{2}|\right)}^{\frac{2}{\tau }}$$
  in which τ=2. This implies that there is a positive constant $\varphi_{1},\varphi_{2}>0$ and a τ>1 such that the integrand component of J satisfies
  $$L\left(I,R_{1},u_{1},u_{2}\right)\leq \varphi_{1}+\varphi_{2}{\left(|u_{1}^{2}\left|+\right|u_{2}^{2}|\right)}^{\frac{2}{\sigma }}.$$

From the above proof, we have that our system satisfies the necessary conditions in Theorem 2, which is completed here. □

### 5.2. Characterization of the Optimal Control Problem

The Pontryagin’s Maximum principle [58] allows us to utilize costate functions to transform the optimization problem to the problem of determining the point-wise minimum of $u_{1}^{*}$ and $u_{2}^{*}$ of the Hamiltonian. This Hamiltonian function is built using the cost function in (2) and the malaria model in (1). With this, we derive:

(28) $$\begin{array}{cc} H(X,\lambda_{j},u_{i})\; & =\omega_{1}u_{1}^{2}+\omega_{2}u_{2}^{2}+\omega_{3}I+\omega_{4}R_{1}\\ \; & +\lambda_{1}\left[\Pi_{h}+\kappa R_{1}+\vartheta R_{2}+\alpha S_{2}-\beta_{h1}S_{1}W-\left(u_{1}\left(t\right)+\mu_{h}\right)S_{1}\right]\\ \; & +\lambda_{2}\left[u_{1}\left(t\right)S_{1}-\beta_{h2}S_{2}W-\left(\alpha +\mu_{h}\right)S_{2}\right]\\ \; & +\lambda_{3}\left[\beta_{h1}S_{1}W-\left(\delta_{1}+\mu_{h}\right)E_{1}\right]\\ \; & +\lambda_{4}\left[\beta_{h2}S_{2}W-\left(\delta_{2}+\mu_{h}\right)E_{2}\right]\\ \; & +\lambda_{5}\left[\delta_{1}E_{1}+\delta_{2}E_{2}+\left(1-p-q\right)\gamma_{2}T-\left(u_{2}\left(t\right)+\gamma_{1}+\mu_{h}\right)I\right]\\ \; & +\lambda_{6}\left[u_{2}\left(t\right)I-p\gamma_{2}T-q\gamma_{2}T-\left(1-p-q\right)\gamma_{2}T-\mu_{h}T\right]\\ \; & +\lambda_{7}\left[\gamma_{1}I+q\gamma_{2}T-\left(\kappa +\mu_{h}\right)R_{1}\right]\\ \; & +\lambda_{8}\left[p\gamma_{2}T-\left(\vartheta +\mu_{h}\right)R_{2}\right]\\ \; & +\lambda_{9}\left[\Pi_{v}-\beta_{v}\left(\zeta_{e1}E_{1}+\zeta_{e2}E_{2}+I+\zeta_{t}T+\zeta_{r}R_{1}\right)V-\mu_{v}V\right]\\ \; & +\lambda_{10}\left[\beta_{v}\left(\zeta_{e1}E_{1}+\zeta_{e2}E_{2}+I+\zeta_{t}T+\zeta_{r}R_{1}\right)V-\mu_{v}W\right], \end{array}$$

where $X=(S_{1},S_{2},E_{1},E_{2},I,T,R_{1},R_{2},V,W)$ and $\lambda_{j}$ for i=1,2,⋯10 are the associated adjoints for the model variables $S_{1},S_{2},E_{1},E_{2},I,T,R_{1},R_{2},V,$ and *W*, respectively.

Next, we will calculate the optimality system of our optimal control problem. The following results is a direct consequences of application of the Pontriagin’s Maximum Principle for bounded controls [59]. The adjoint system can be written as follows:

(29) $$\begin{array}{cc} \frac{d\lambda_{1}}{dt} & =-\frac{\partial L}{\partial S_{1}}\\ & =\beta_{h1}W\left(\lambda_{1}-\lambda_{3}\right)+u_{1}\left(\lambda_{1}-\lambda_{2}\right)+\mu_{h}\lambda_{1},\\ \frac{d\lambda_{2}}{dt} & =-\frac{\partial L}{\partial S_{2}}\\ & =\beta_{h2}W\left(\lambda_{2}-\lambda_{4}\right)+\alpha \left(\lambda_{2}-\lambda_{1}\right)+\mu_{h}\lambda_{2},\\ \frac{d\lambda_{3}}{dt} & =-\frac{\partial L}{\partial E_{1}}\\ & =\delta_{1}\left(\lambda_{3}-\lambda_{5}\right)+\zeta_{e1}\beta_{v}V\left(\lambda_{9}-\lambda_{10}\right)+\mu_{h}\lambda_{3},\\ \frac{d\lambda_{4}}{dt} & =-\frac{\partial L}{\partial E_{2}}\\ & =\delta_{2}\left(\lambda_{4}-\lambda_{5}\right)+\zeta_{e2}\beta_{v}V\left(\lambda_{9}-\lambda_{10}\right)+\mu_{h}\lambda_{4},\\ \frac{d\lambda_{5}}{dt} & =-\frac{\partial L}{\partial I}\\ & =-\omega_{3}+u_{2}\left(\lambda_{5}-\lambda_{6}\right)+\gamma_{1}\left(\lambda_{5}-\lambda_{7}\right)+\beta_{v}V\left(\lambda_{9}-\lambda_{10}\right)+\mu_{h}\lambda_{5},\\ \frac{d\lambda_{6}}{dt} & =-\frac{\partial L}{\partial T}\\ & =\gamma_{2}\left(\lambda_{6}-\lambda_{5}\right)+p\gamma_{2}\left(\lambda_{5}-\lambda_{8}\right)+q\gamma_{2}\left(\lambda_{5}-\lambda_{7}\right)+\zeta_{t}\beta_{v}V\left(\lambda_{9}-\lambda_{10}\right)+\mu_{h}\lambda_{6},\\ \frac{d\lambda_{7}}{dt} & =-\frac{\partial L}{\partial R_{1}}\\ & =-\omega_{4}+\kappa \left(\lambda_{7}-\lambda_{1}\right)+\zeta_{r}\beta_{v}V\left(\lambda_{9}-\lambda_{10}\right)+\mu_{h}\lambda_{7},\\ \frac{d\lambda_{8}}{dt} & =-\frac{\partial L}{\partial R_{2}}\\ & =\vartheta \left(\lambda_{8}-\lambda_{1}\right)+\mu_{h}\lambda_{8},\\ \frac{d\lambda_{9}}{dt} & =-\frac{\partial L}{\partial V}\\ & =\beta_{v}\left(\zeta_{e1}E_{1}+\zeta_{e2}E_{2}+I+\zeta_{t}T+\zeta_{r}R_{1}\right)\left(\lambda_{9}-\lambda_{10}\right)+\mu_{v}\lambda_{9},\\ \frac{d\lambda_{10}}{dt} & =-\frac{\partial L}{\partial W}\\ & =\beta_{h1}S_{1}\left(\lambda_{1}-\lambda_{3}\right)+\beta_{h2}S_{2}\left(\lambda_{2}-\lambda_{4}\right)+\mu_{v}\lambda_{10}, \end{array}$$

and with transversality conditions $\lambda_{j}\left(T\right)=0$ for j=1,2,⋯10. The optimality conditions requires that $\frac{\partial H}{\partial u_{1}}=\frac{\partial H}{\partial u_{2}}=0$. Hence, according to our model and cost function, and following the lower and upper bounds, we have:

(30) $$\begin{array}{cc} u_{1}^{*} & =\min\left\{\max\left\{0,\frac{S_{1}\left(\lambda_{2}-\lambda_{1}\right)}{2\omega_{1}}\right\},1\right\}, \end{array}$$

(31) $$\begin{array}{cc} u_{2}^{*} & =\min\left\{\max\left\{0,\frac{I(\lambda_{6}-\lambda_{5})}{2\omega_{2}}\right\},1\right\}. \end{array}$$

## 6. Numerical Simulation of the Optimal Control Problem

From the previous analysis, our optimal control problem consists of the state system, which is the malaria model in (1) with initial condition X(0), the costate system in (29) with transversality condition $\lambda_{j}\left(T\right)=0$, and optimal conditions in (30). To determine the optimal trajectory of $u_{1}^{*}$ and $u_{2}^{*}$, we have to determine the solution of the state and costate system. Since the state system has an initial condition, while the costate has the final condition, we cannot solve these systems directly forward in time. Thus, we will solve it using the “forward-backward sweep method” [59]. The algorithm is as follows. An initial guess for the control variables should be made. Then, with this initial guess, we solve the state system in (1) numerically forward in time using a Runge–Kutta method. Having the initial guess of control variables and the solution of the state system for t∈[0,T], we solve our costate system in (29) backward in time, also with the Runge–Kutta method. Having the solution of state and costate for t∈[0,T], we update the control trajectories using the equation in (30). This process is repeated until the convergence criteria are satisfied. In our numerical implementation, the convergence criteria are when the Euclidean distance between $i^{th}$ and ${\left(i+1\right)}^{th}$ iteration for costate, state, and control parameters are smaller than our chosen tolerance.

Unless stated otherwise, to run the simulations in this section, we used parameter values as shown in Table 1, with the following initial conditions: $S_{1}\left(0\right)=95\%N,S_{2}\left(0\right)=0,E_{1}\left(0\right)=1\%N,E_{2}\left(0\right)=0,I\left(0\right)=3\%N,T=0,R_{1}\left(0\right)=0,R_{2}\left(0\right)=1\%N,V\left(0\right)=180\%N,$ and W(0)=20%N, with *N* is the total of human population in Papua (3,435,430). The values assigned for the weight constant are $\omega_{1}=1,\omega_{2}=1,\omega_{3}=5\times 10^{11},$ and $\omega_{4}=10^{12}$. In this section, the impact of control trajectories with various scenarios is studied. We studied the impact on the change of the dynamic of each sub-population (with and without controls), the total of susceptible and infected populations, and the cost related to the scenario.

### 6.1. Different Combination of Interventions

**Figure 8:**
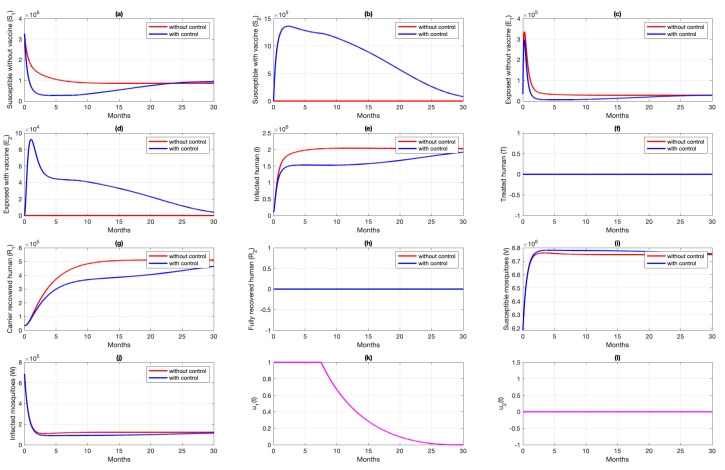
Dynamics of human (**a**–**h**), mosquitoes (**i**,**j**), and controls (**k**,**l**) under the scenario of pre-erythrocytic vaccine intervention only applies. Blue and red curve represents implementation strategies with and without controls.

**Figure 9:**
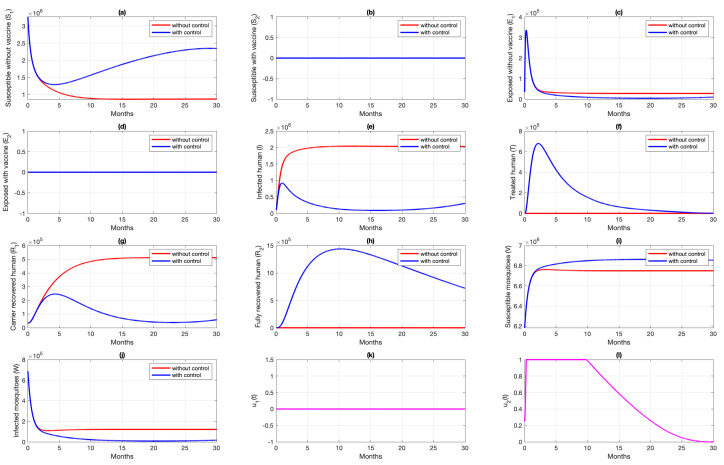
Dynamics of human (**a**–**h**), mosquitoes (**i**,**j**), and controls (**k**,**l**) under the scenario of transmission-blocking treatment intervention only applies. Blue and red curve represents implementation strategies with and without controls.

**Figure 10:**
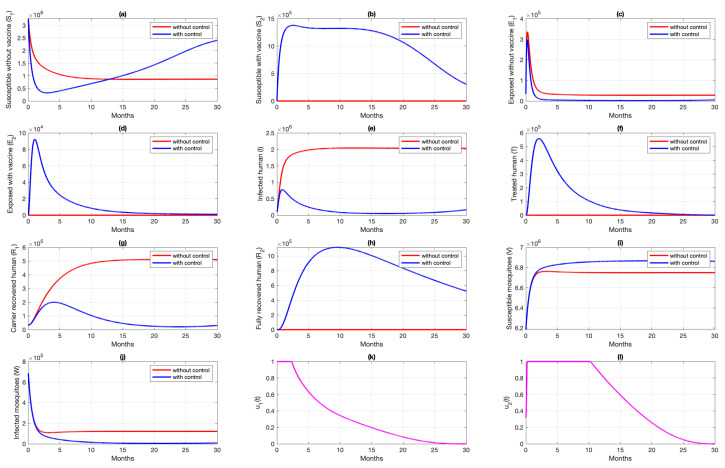
Dynamics of human (**a**–**h**), mosquitoes (**i**,**j**), and controls (**k**,**l**) under the scenario of both intervention applies. Red and blue curve present a condition of without and with implementation of control.

**Figure 11:**
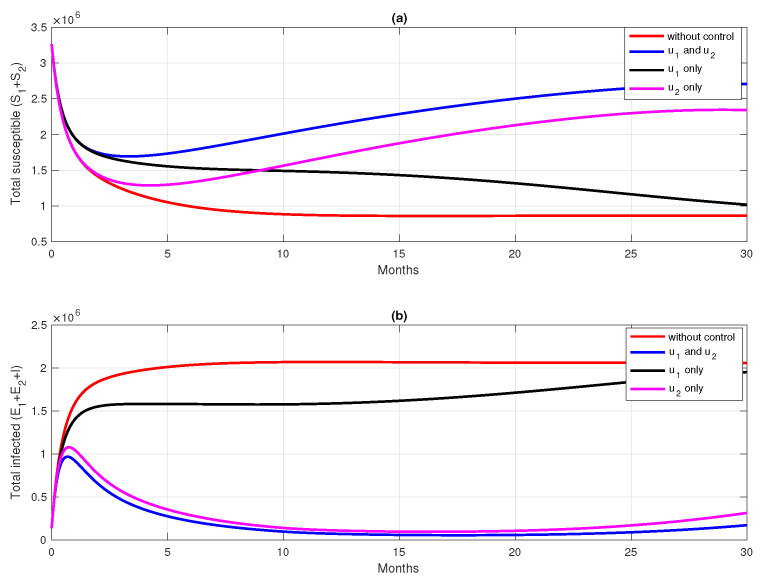
Dynamics of total susceptible human (**a**) total of infected human (**b**) for four different strategies (no intervention (red), $u_{1}$ and $u_{2}$ (blue), $u_{1}$ only (black), and $u_{2}$ only (magenta)).

For the first scenario of optimal control simulations, we conducted our experiment based on the combination of controls that have been used. Let Scenarios 1, 2, and 3 be scenarios when only pre-erythrocytic drugs are used $\left(u_{1}>0,u_{2}=0\right)$, transmission-blocking drugs are used $\left(u_{1}=0,u_{2}>0\right)$, and both drugs are used $\left(u_{1}>0,u_{2}>0\right)$, respectively. The illustration on the dynamics of system (1) and the control profiles for Scenarios 1, 2, and 3 are given in Figure 8, Figure 9, and Figure 10, respectively. From Figure 8, we can see that with a profile of $u_{1}$ of Scenario 1 in Figure 8k, the number of infected individuals is always decreasing except $E_{2}$, as a consequence of the existence of individuals who are already vaccinated $\left(S_{2}\right)$. It is clearly observed that the pre-erythrocytic vaccine should be maintained at one from the start of the simulation for a long time period to increase the number of vaccinated individuals. With a large number of vaccinated individuals, fewer people will be infected by mosquitoes. Hence, the total number of infected individuals is always smaller than when no intervention is used. The total cost for Scenario 1 is $4.31889\times 10^{12}$, with an average of total avoided infected individuals $\left(E_{1}+E_{2}+I+T\right)$ compared to without intervention scenario is 34,679 individuals. Please see Figure 11 for the dynamics of total susceptible and infected individuals, with and without control.

Simulation results of Scenario 2, when only transmission-blocking is used as an intervention strategy, are given in Figure 9. Similar to Scenario 1, the intervention of transmission-blocking successfully reduces the number of infected individuals significantly. The trajectories of $u_{2}$ for Scenario 2 can be seen in Figure 9l, where intervention should be given in its maximum effort from the beginning of simulation for 10 months and decreasing slowly as time passes. The total cost for Scenario 2 is $1.33452\times 10^{12}$, which is almost 70% smaller than Scenario 1. The average number of total infected inverted in Scenario 2 is 160,114 individuals. Compared to Scenario 1, the average number of averted cases for Scenario 2 is almost 361% larger.

Figure 10 shows the dynamics of each subpopulation in system (1), and it controls trajectories for Scenario 3. It reflects the success of the combination between the pre-erythrocytic vaccine and transmission-blocking treatment. This scenario suggests the intervention of transmission-blocking should be given in its maximum effort, much longer than the pre-erythrocytic vaccine. With the total cost for Scenario 3 being $1.334401\times 10^{12}$, the average number of avoided infections is 170,680 individuals.

The comparison of all scenarios in one figure can be seen in Figure 11. We can see that reducing the number of infected individuals between Scenarios 2 and 3 is almost similar, which is more successful than Scenario 1. Hence, with the set of parameters and initial conditions in this article, we can conclude that transmission-blocking treatment is more significant in reducing the number of infected individuals than the pre-erythrocytic vaccine. We have to state that the estimation of parameters on our model does not include the number of individuals who already use both forms of intervention. Hence, it is important to do another parameter estimation when the data for the number of individuals who use both interventions are already available and re-simulate our optimal control simulation.

### 6.2. Different Initial Condition of Population

**Figure 12:**
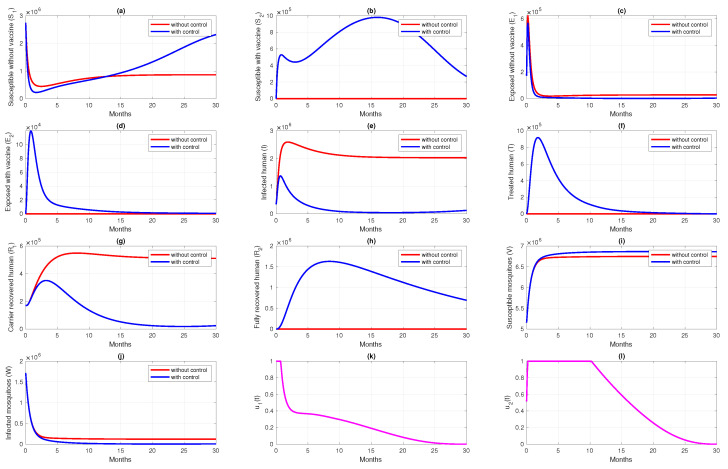
Dynamics of human (**a**–**h**), mosquitoes (**i**,**j**), and controls (**k**,**l**) under the scenario of both intervention applies, but with a higher number of infected individuals at time t=0. Red and blue curve present a condition of without and with implementation of control.

In this subsection, we analyze the impact of the initial condition of the population on the dynamics of control variables. We use parameter values and initial condition of Scenario 3 in Section 6.1 as the base case, and change the initial conditions as follows: $S_{1}\left(0\right)=80\%N,S_{2}\left(0\right)=0,E_{1}\left(0\right)=5\%N,E_{2}\left(0\right)=0,I\left(0\right)=10\%N,T=0,R_{1}\left(0\right)=0,R_{2}\left(0\right)=5\%N,V\left(0\right)=150\%N,$ and W(0)=50%N, with *N* being the total human population in Papua (3,435,430). Let us call this Scenario 4. In Scenario 4, we can see that the number of infected individuals in the human and mosquito population is larger than in Scenario 3. The result of this scenario is shown in Figure 12. We can see in Figure 12k that the dynamics of $u_{1}$ are only given in its maximum effort for just a few days of simulation, and start to decrease rapidly compared to in Scenario 3 (Please see Figure 10k). Since the rate of vaccine is the maximum given in a concise time period, then the increased number of $S_{2}$ in Scenario 4 is not as significant as in Scenario 3. The dynamics of $u_{2}$, which present the transmission-blocking treatment, should be given its maximum value over a longer period than $u_{1}$ to reduce the number of infected individuals. Consequently, the number of treated individuals increases very rapidly and decreases as they begin to recover. The cost for Scenario 4 is $4.6189\times 10^{12}$, which is much larger than Scenario 3. Although the number of infected cases avoided in Scenario 4 is 1,177,307, which is more significant than in Scenario 3, the cost to achieve this result is almost 330% larger than in Scenario 4. Hence, we can conclude that when the number of infected individuals is relatively large at the beginning of the intervention period, most of the intervention should be focused on reducing cases, which in our model is the implementation of transmission-blocking treatment. With more infected individuals at the beginning of the intervention period, more costs are needed to control the spread of malaria.

### 6.3. Different Initial Basic Reproduction Number

In this subsection, the optimal control simulation aims to see the effect of the initial value of the basic reproduction number when pre-erythrocytic intervention and transmission-blocking treatment is not yet implemented. In this case, the basic reproduction number of system (1) is given by (12).

**Figure 13:**
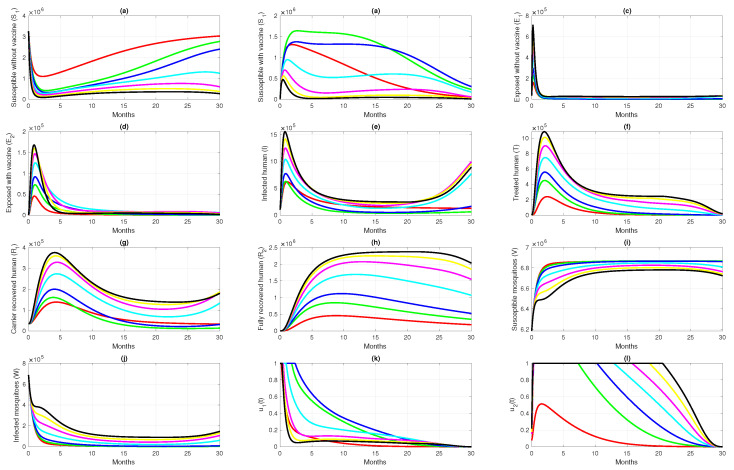
Time series dynamics for; humans (**a**–**h**), mosquitoes (**i**,**j**), and controls (**k**,**l**), but different initial $R_{0}$ when no control applied. The color in the legend are explained in Table 5.

For this simulation, we used parameter values as from Table 1, except that $u_{1}\;=\;0,u_{2}=0$, and also other parameters that related to the control parameters are zero as the base case (case 3 in Table 5). We run our simulation in seven different cases, and the differences are based on the value of $\beta_{h1},\beta_{h2},$ and $\beta_{v}$. Hence, we also have a different initial basic reproduction number $R_{0}$, as shown in Table 5. It can be seen that the greater the value of $R_{0}$, the higher the costs to reduce the high number of cases in the field. If we pay attention to Figure 13k,l, the higher the $R_{0}$ value, the longer the transmission-blocking treatment intervention should be given to its maximum value, and at the same time, the pre-erythrocytic intervention should be reduced in intensity to avoid high intervention costs. On the other hand, when $R_{0}$ is smaller, the pre-erythrocytic vaccine should be given for a longer period at its maximum level to avoid an increase in the number of malaria-infected individuals.

## 7. Conclusions

In this paper, we formulated and analyzed a deterministic compartmental model for malaria transmission. The model consists of eight compartments for humans and two compartments for mosquitoes. The malaria-free equilibrium is locally asymptotically stable if the basic reproduction number is less than unity. The basic reproduction number of our model presents the combination of infection paths in our transmission model, such as the bite of susceptible mosquitoes to the exposed human (with/without the vaccine), infected, treated, or carrier recovered individuals, or the bite of infected mosquitoes to susceptible humans (with/without vaccine).

Our model parameter values were estimated using incidence data from the Papua and West Papua provinces in Indonesia. We found that without implementing any type of intervention, the basic reproduction number of malaria in Papua and West Papua is greater than one, which suggests that a massive intervention should be made to reduce the spread of malaria. Our sensitivity analysis of the basic reproduction number shows that transmission-blocking is more sensitive to reducing the basic reproduction number compared to the pre-erythrocytic vaccine.

Several scenarios on the optimal control simulation were carried out based on the combination of interventions (first scenario), different initial conditions (second scenario), and the value of the basic reproduction number (third scenario). From the first simulation, in order to understand the most effective combination strategies, we found whether using both vaccine and treatment as a single or combined form of intervention will be more effective compared to other possible combinations. Our analysis shows that using both interventions is the most successful strategy in reducing the number of new infections. However, the number of averted infections with this strategy is only slightly different compared to that of implementing transmission-blocking treatment as a single form of intervention, but with a cheaper cost of implementation. On the other hand, the pre-erythrocytic vaccine is not recommended as a single form of intervention, as the result in reducing the number of infected individuals is not as significant as other strategies. From the second scenario, we concluded that more cost is needed to control malaria when the number of infected individuals is already high. For the last scenario, we run our simulation for various values of the basic reproduction number to indicate that the level of endemicity of malaria when vaccines and treatment are not yet implemented. Our results suggest the larger the basic reproduction number of an area, the larger the scale of the implementation of transmission-blocking treatment is needed to reduce the spread of malaria. Additionally, the pre-erythrocytic vaccine should be implemented at its maximum rate for a short period to minimize the intervention cost.

## Figures and Tables

**Table 1:** The parameters of malaria model in (1). Some parameter values are taken from parameter estimation results in Section 2.2 and the rest are from assumptions.

Symbols	Biological Definitions	Papua	West Papua	Sources
$\Pi_{h}$	Natural birth rate of humans	$\frac{3,435,430}{71.5\;\times \;12}$	$\frac{981,822}{71.5\;\times \;12}$	[32,33,34]
$\Pi_{v}$	Natural birth rate of mosquitos	$\frac{2\;\times \;3,435,430}{\frac{21}{30}}$	$\frac{2\;\times \;981,822}{\frac{21}{30}}$	[32,33,35]
*b*	The average number of mosquitoes bite per unit time	9.07523	5.94285	estimated
$\bar{\beta_{h1}}$	The successful transmission rate of susceptible humans per bite	0.89999	0.87753	estimated
$\bar{\beta_{h2}}$	The successful transmission rate of susceptible humans who receive pre-erythrocytic vaccine per bite	0.42299	0.41244	estimated
$\beta_{h1}=\frac{b\bar{\beta_{h1}}}{N}$	Average infection rate to humans per unit time per mosquito	$2.37749\;\times \;10^{-6}$	$5.31162\;\times \;10^{-6}$	estimated
$\beta_{h2}=\frac{b\bar{\beta_{h2}}}{N}$	Average infection rate to humans who receive pre-erythrocytic vaccine per unit time per mosquito	$1.11742\;\times \;10^{-6}$	$2.49646\;\times \;10^{-6}$	estimated
$\bar{\beta_{v}}$	Average of successful transmission rate of susceptible mosquitos per bite	0.00572	0.01054	estimated
$\beta_{v}=\frac{b\bar{\beta_{v}}}{N}$	Average infection rate to mosquitos per unit time per human	$1.51190\;\times \;10^{-8}$	$6.38376\;\times \;10^{-8}$	estimated
κ	Waning rate of temporal immunity from recovered carriers	0.33333	0.33333	estimated
ϑ	Waning rate of temporal immunity from fully recovered class	0.05555	0.05555	estimated
$u_{1}$	Vaccination rate with pre-erythrocytic vaccine	0.001	0.001	assumption
$u_{2}$	Rate of treatment with transmission-blocking drugs	0.499999	0.499997	estimated
1−ξ	pre-erythrocytic vaccine efficacy level	0.53	0.53	[36]
α	Waning rate of vaccine efficacy	$\frac{1}{6}$	$\frac{1}{6}$	[37]
$\delta_{1}$	Progression rate from exposed without vaccine class	6.08333	6.08333	estimated
$\delta_{2}$	Progression rate from exposed with vaccine class	2	2	[36,38]
$\gamma_{1}$	Natural recovery rate of infected humans by immune response	0.19999	0.19999	estimated
$\gamma_{2}^{-1}$	Duration of treatment with transmission-blocking drugs	$\frac{1}{1.08631}$	$\frac{1}{1.08631}$	estimated
*p*	Proportion of people in treatment who managed to get protection	0.59999	0.59964	estimated
*q*	Proportion of people in treatment who fail to receive protection and then recover naturally	0.29999	0.29994	estimated
1−p−q	Proportion of people in treatment who fail to receive protection and then return to the infected class	0.100000004	0.10040	estimated
$\zeta_{e1}$	Correction factor for infection rate in mosquitoes by exposed humans	0.04999	0.04999	estimated
$\zeta_{e2}$	Correction factor for infection rate in mosquitoes by exposed humans with vaccine	0.03999	0.03999	assumption
$\zeta_{r}$	Correction factor for infection rate in mosquitoes by recovered humans but carrier	0.06	0.06	estimated
$\zeta_{t}$	Correction factor for infection rate in mosquitoes by humans in treatment	0.08	0.08	estimated
$\mu_{h}$	Natural death rate of humans	$\frac{1}{71.5\;\times \;12}$	$\frac{1}{71.5\times 12}$	[34]
$\mu_{v}$	Natural death rate of mosquitos	$\frac{30}{21}$	$\frac{30}{21}$	[35]

**Table 2:** Lower and upper bound of parameters that will be estimated.

Parameters	Interval Values	Sources
$\Pi_{h}$	$\frac{3,435,430}{71.5\times 12}$ or $\frac{981,822}{71.5\times 12}$	[32,33,34]
$\Pi_{v}$	$2\times 3,435,430\times \frac{30}{21}$ or $2\times 981,822\times \frac{30}{21}$	[32,33,35]
*b*	$\left[3.012,17.4\right]$	[27]
$\bar{\beta_{h1}}$	$\left[0.03,0.9\right]$	[16,27]
$\bar{\beta_{h2}}$	$\left[0.47\times 0.03,0.47\times 0.9\right]$	[16,27,36]
$\beta_{h1}=\frac{b\bar{\beta_{h1}}}{N}$	$\left[2.63\times 10^{-8},4.22\times 10^{-6}\right]$ or $\left[9.20\times 10^{-8},1.47\times 10^{-5}\right]$	[27,32,33]
$\beta_{h2}=\frac{b\bar{\beta_{h2}}}{N}$	$\left[1.23\times 10^{-8},1.98\times 10^{-6}\right]$ or $\left[4.32\times 10^{-8},6.94\times 10^{-6}\right]$	[27,32,33,36]
$\bar{\beta_{v}}$	$\left[0.00572,0.09\right]$	[27]
$\beta_{v}=\frac{b\bar{\beta_{v}}}{N}$	$\left[5.01\times 10^{-9},4.55\times 10^{-7}\right]$ or $\left[1.75\times 10^{-8},1.59\times 10^{-6}\right]$	[27,32,33]
κ	$\left[\frac{1}{12},\frac{1}{3}\right]$	[38]
ϑ	$\left[\frac{1}{18},\frac{1}{3}\right]$	[38]
$u_{1}$	$\left(0,1\right)$	varied
$u_{2}$	$\left(0,1\right)$	varied
1−ξ	0.53	[36]
α	$\left[\frac{1}{4\times 12},\frac{1}{4}\right]$	[37]
$\delta_{1}$	$\left[2.02778,6.08334\right]$	[38]
$\delta_{2}$	$\left[0.9530566,2.8591698\right]$	[36,38]
$\gamma_{1}$	$\left[\frac{1}{12},\frac{1}{5}\right]$	[46]
$\gamma_{2}$	$\left[0.7242071429,1.086310714\right]$	[47]
*p*	$\left[0,1\right]$	varied
*q*	$\left[0,1\right]$	varied
1−p−q	$\left[0,1\right]$	varied
$\zeta_{e1}$	$\left[0.00001,1\right)$	[16]
$\zeta_{e2}$	$\left(0,1\right)$	varied
$\zeta_{r}$	$\left[0.005,1\right)$	[16]
$\zeta_{t}$	$\left[0.02,1\right)$	[16]
$\mu_{h}$	$\frac{1}{71.5\times 12}$	[34]
$\mu_{v}$	$\frac{30}{21}$	[35]

**Table 3:** Parameter values to show existence of $E^{†}$.

Parameter	Value	Parameter	Value
κ	0.333333327466586	$\gamma_{1}$	0.199999993947735
ϑ	0.0555555854358188	$\gamma_{2}$	1.08631070229205
α	$\frac{1}{6}$	$\beta_{v}$	$1.51190029406934\times 10^{-8}$
$\beta_{h1}$	$2.37749246426963\times 10^{-6}$	$\zeta_{e1}$	0.0499997440960968
$u_{1}$	0.001	$\zeta_{e2}$	0.0399997440960968
$\beta_{h2}$	$1.11742145820672\times 10^{-6}$	$\zeta_{t}$	0.080000001876738
$\delta_{1}$	6.08333998105411	$\zeta_{r}$	0.060000000761188
$\delta_{2}$	2	$\mu_{h}$	$1.165501166\times 10^{-3}$
*p*	0.599999998192909	$\Pi_{h}$	4003.99766899767
*q*	0.299999997668467	$\mu_{v}$	1.428571429
$u_{2}$	0.499999999825239	$\Pi_{v}$	9,815,514.28571429

**Table 4:** PRCC parameter values and *p*-values for the global sensitivity analysis against $R_{0}$.

Parameter	PRCC Values	*p*-Values	Significant?
$\Pi_{h}$	0.271036046	0	TRUE
$\beta_{h1}$	0.631568463	0	TRUE
$\mu_{h}$	−0.656060428	0	TRUE
$\mu_{v}$	−0.856948705	0	TRUE
$\beta_{v}$	0.643359162	0	TRUE
$\xi_{e1}$	0.038340724	$2.282\times 10^{-1}$	FALSE
$\Pi_{v}$	0.276430743	0	TRUE
$\delta_{1}$	0.003574822	$9.106\times 10^{-1}$	FALSE
$\gamma_{1}$	−0.162743705	$2.486\times 10^{-7}$	TRUE
κ	−0.079425150	$1.240\times 10^{-2}$	FALSE
$\xi_{r}$	0.183579610	$5.425\times 10^{-9}$	TRUE

**Table 5:** Numerical results for various initial value of the non-controlled basic reproduction number.

Case	Scenario for $\beta_{h1}$, $\beta_{h2}$ and $\beta_{v}$	$R_{0}$	Inverted Case	Cost	Colour
1	Reduced 50% from case 3	1.005	52,787	$7.038\times 10^{11}$	Red
2	Reduced 25% from case 3	1.508	129,702	$1.101\times 10^{12}$	Green
3	As in Table 1 for Papua data	2.011	170,680	$1.334\times 10^{12}$	Blue
4	Increased 50% from case 3	3.016	185,168	$1.857\times 10^{12}$	Cyan
5	Increased 100% from case 3	4.022	183,584	$2.67\times 10^{12}$	Magenta
6	Increased 150% from case 3	5.027	184,294	$3.573\times 10^{12}$	Yellow
7	Increased 200% from case 3	6.033	185,505	$4.562\times 10^{12}$	Black

## Data Availability

The data that been used in this research is from personal request to Papua and West Papua Provincial Health Office.
